# Designing a novel multiepitope vaccine candidate against *Treponema pallidum* vi*a* adhesins using reverse vaccinology

**DOI:** 10.1038/s41598-026-45084-1

**Published:** 2026-04-01

**Authors:** Hongmei Tang, Zhixi Chen, Hongxia Yan, Zhen He, Ranhui Li, Yafeng Xie, Xiaoliu Wang

**Affiliations:** 1 Department of Dermatology and Venereology, The First Affiliated Hospital, Hengyang Medical College, University of South China, Hengyang, 421001 Hunan China; 2 Department of Clinical Laboratory, The Second Affiliated Hospital, Hengyang Medical School, University of South China, Hengyang, 421001 Hunan China; 3 Department of Blood Transfusion, The First Affiliated Hospital, Hengyang Medical College, University of South China, Hengyang, 421001 Hunan China; 4 Department of Pediatrics, The Second Affiliated Hospital, Hengyang Medical School, University of South China, Hengyang, 421001 Hunan China; 5 Institute of Pathogenic Biology, Basic Medical School, Hengyang Medical College, Key Laboratory of Special Pathogen Prevention and Control of Hunan Province, University of South China, Hengyang, 421001 China

**Keywords:** Syphilis, Adhesins, *Treponema pallidum*, Multiepitope vaccine, Reverse vaccinology, Biotechnology, Computational biology and bioinformatics, Immunology, Microbiology

## Abstract

Syphilis, caused by *Treponema pallidum* (*T. pallidum*), represents a significant worldwide public health threat. This spiral-shaped, Gram-negative pathogen is a strict human-specific obligate parasite primarily transmitted through sexual contact. This pathogen induces a multistage and multisystem progressive disease, against which no effective prophylactic vaccine currently exists. This study focuses on syphilis prevention and control by employing a reverse vaccinology approach to investigate the immunogenic properties of *T. pallidum* adhesin proteins. Fifteen T-cell epitopes and seven B-cell epitopes were screened and linked in series using appropriate linkers to construct a multi-epitope vaccine. The vaccine was subjected to in silico analysis, including secondary and tertiary structure prediction, molecular docking, and molecular dynamics simulation. Based on these analyses, a recombinant plasmid, pET-28a(+)-MEVTP, was constructed, and the purified recombinant protein was obtained via nickel column affinity chromatography. In silico immune simulation results suggested that the vaccine could induce specific cellular and humoral immune responses. However, further experimental evaluation of its immunological effects is required to validate the computationally predicted immunogenicity, thereby establishing an experimental basis for its advancement toward translational medical applications and providing critical evidence to support syphilis prevention and control efforts.

**Supplementary Information:**

The online version contains supplementary material available at 10.1038/s41598-026-45084-1.

## Introduction

Syphilis is a lasting systemic sexually transmitted infection brought about by *Treponema pallidum* (*T. pallidum*). Annually, new cases of adult infections are reported, and continue to pose a significant challenge to global public health^1–3^. The pathogenicity of syphilis critically depends on the invasive capacity of *T. pallidum* and its remarkable mechanisms for immune evasion^4^. The bacterium adheres to host cells and extracellular matrix components, facilitating its proliferation and triggering inflammatory responses. Although infected individuals mount immune responses, *T. pallidum* successfully evades immune clearance through mechanisms such as antigenic variation and virulence variation, which enable its persistent survival within the host and contribute to the recurrence of disease progression.

Bacterial adhesins are a class of proteins or polysaccharide complexes located on the bacterial surface that specifically recognize and bind to receptors on host cell surfaces or extracellular matrix components, facilitating bacterial colonization and infection within the host^5^. Treponemal adhesins represent a specialized class of proteins on the surface of *T. pallidum*, exhibiting characteristics analogous to bacterial adhesins. Functioning as virulence determinants of *T. pallidum*, these adhesin proteins participate in the infection process by mediating host adhesion, tissue invasion, and immune evasion^6,7^. While possessing a conserved C-terminal transmembrane domain and a functional N-terminal epitope, these proteins demonstrate significant molecular heterogeneity. In terms of binding specificity, Tp0136 preferentially binds cellular fibronectin; Tp0155 and Tp0483 recognize fibronectin; Tp0435 promotes cellular adhesion; Tp0750 uses its metal ion-dependent adhesion site to bind fibrinogen and Annexin A2 protein; Tp0751 exhibits broad-spectrum binding to laminin isoforms; and Tp0954 mediates dissemination dependent on placental glycosaminoglycans. Structural diversity is manifested in the β-barrel basin domain of Tp0435, the von Willebrand factor type A domain of Tp0750, and the binary topology of Tp0751^8^.

The prolonged asymptomatic latency and insidious clinical manifestations of syphilis enable untreated individuals to serve as long-term reservoirs, thereby exacerbating transmission dynamics. The natural progression of syphilis is characterized by three distinct stages: (1) primary syphilis, characterized by painless genital ulceration (chancre) developing two to four weeks post-infection; (2) secondary syphilis, during which systemic manifestations, including diffuse cutaneous rash and lymphadenopathy, emerge weeks to months later; and (3) tertiary syphilis, in which untreated individuals develop severe complications years later, affecting visceral organs, the skeletal system, and the nervous system, leading to cardiovascular syphilis, neurosyphilis, and other irreversible, destructive pathologies^9,10^. Currently, penicillin remains the first-line therapy for syphilis; however, its limitations are becoming increasingly evident. Patients with neurosyphilis and congenital syphilis require prolonged intravenous administration for up to 10 to 14 days^11,12^. For penicillin-allergic pregnant individuals, therapeutic options are severely limited. Doxycycline, a commonly prescribed antibiotic, is contraindicated due to its teratogenic risks and potential for placental transmission. Consequently, there is a critical lack of safe and effective alternative therapeutic options for this vulnerable population^13^.

The creation of vaccines for syphilis remains at the preclinical and foundational phases, encountering numerous obstacles. *T. pallidum* possesses a limited array of outer membrane proteins, with certain proteins, such as the Tpr protein family, exhibiting significant antigenic variation^14^. Moreover, individuals remain susceptible to reinfection by heterologous strains^15^. To date, several vaccine candidates have been investigated, including *T. pallidum* outer membrane proteins, flagellar proteins, heat shock proteins (HSPs), DNA vaccines, and live attenuated vaccines^14,16–18^. Among these, Tp0751 and the flagellar protein FlaB3 have shown the capacity to induce protective antibodies; however, *T. pallidum* displays considerable strain-to-strain variability in both antigenic profiles and virulence^19,20^. Consequently, future vaccine candidates must demonstrate broad efficacy against diverse *T. pallidum* strains^21^.

The challenging in vitro culture of *T. pallidum* significantly impedes conventional vaccine development approaches^14^. Reverse vaccinology addresses these limitations by analyzing genomic sequences and proteomic data to predict conserved antigens, offering several advantages^22^. Firstly, it overcomes the reliance on in vitro cultivation by bypassing the need for pathogen culture, which significantly accelerates the vaccine development timeline. Secondly, it addresses antigenic diversity by screening conserved epitopes through genomic alignment and integrating multivalent vaccine design, thereby enhancing cross-protection against diverse strains. Lastly, it improves the feasibility of antigen expression through structural biology-guided optimization of antigen conformation^23–25^. For instance, linking multi-epitopes via flexible or rigid linkers preserves the immunogenicity of native epitope configurations^26,27^. The meningococcal serogroup B vaccine exemplifies the successful realization of reverse vaccinology technology, having received initial regulatory approval in 2014^28,29^.

In this study, we employed a reverse vaccinology approach to screen seven adhesin proteins from *T. pallidum*, identifying seven B-cell epitopes, eleven cytotoxic T lymphocyte (CTL) epitopes, and four helper T lymphocyte (HTL) epitopes. These epitopes constitute a multi-epitope vaccine against *T. pallidum* (MEVTP). Following this, based on the identified immunodominant epitopes, we systematically evaluated and optimized the physicochemical properties, antigenicity, and structural stability of MEVTP using bioinformatics tools. This effort led to the development and formulation of an innovative multi-epitope vaccine candidate, which was purified by Ni^2^⁺ affinity chromatography.

## Methods

**Fig. 1:**
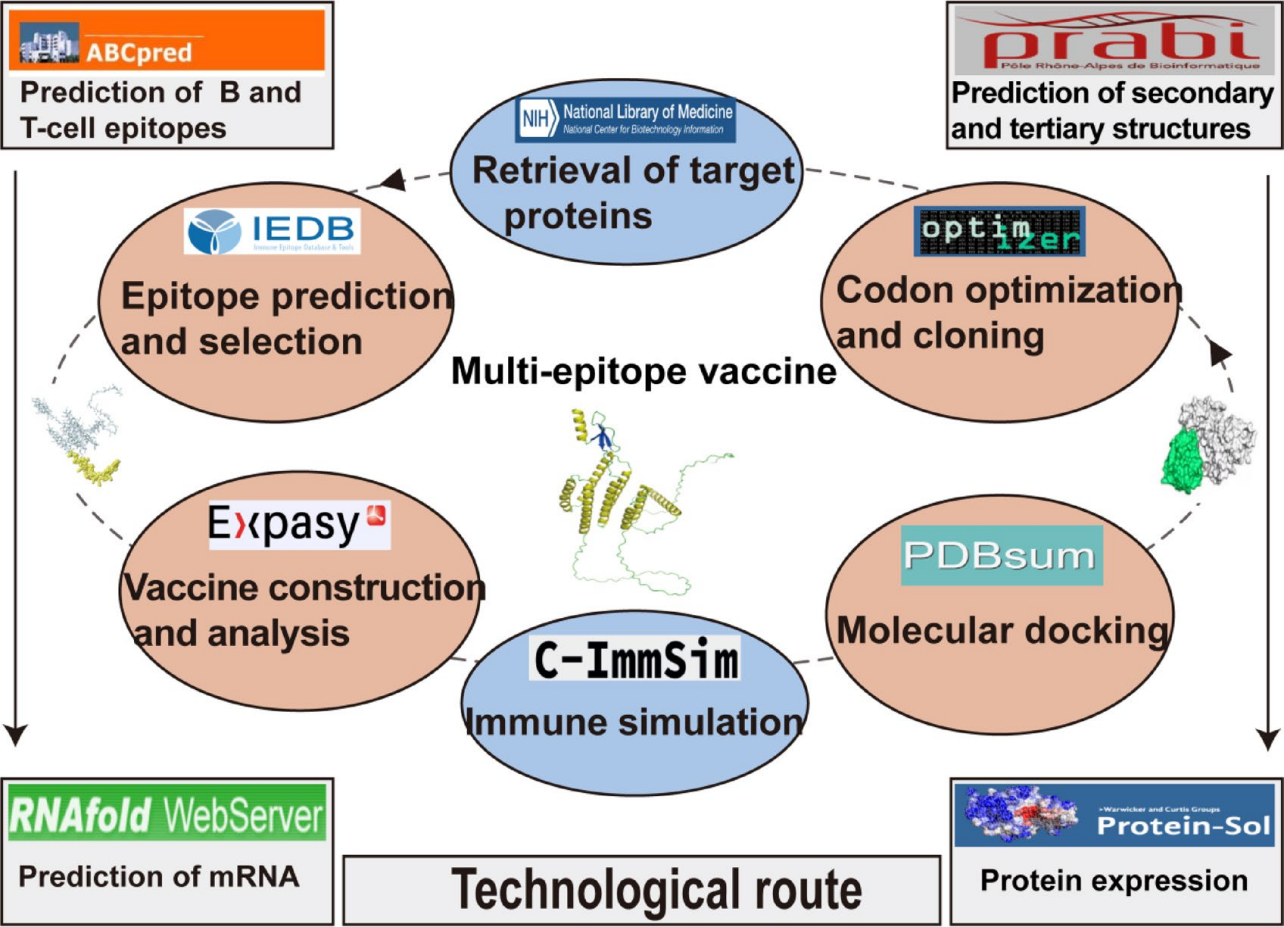
A typical reverse vaccinology workflow.

For the design of this multi-epitope vaccine, we adhered to the established standard protocol reported in previous studies^30^. The workflow is shown in Fig. 1. All URLs for the prediction tools are listed in Supplementary Table 1.

### Acquisition of proteins

Amino acid sequences for the adhesin proteins of *T. pallidum* (Nichols strain) were downloaded in FASTA format from the NCBI database (GenBank: AE000520.1). Seven adhesin proteins were selected: Tp0136 (Protein ID: AAC65137.1), Tp0155 (Protein ID: AAC65145.1), Tp0435 (Protein ID: AAC65423.1), Tp0483 (Protein ID: AAC65472.1), Tp0750 (Protein ID: AAC65716.1), Tp0751 (Protein ID: AAC65720.1), and Tp0954 (Protein ID: AAC65909.1). The analysis of the physicochemical characteristics of the selected adhesins was conducted, with a focus on factors including antigenicity, molecular weight, solubility, instability index, and theoretical isoelectric point. All candidate proteins were subjected to homology alignment via BLASTp on NCBI to reduce the risk of autoimmune responses. Human (taxid: 9606) and mouse (taxid: 10,088) were input into the Organism Optional field, with a sequence identity of < 30% selected^31^.

### Epitope prediction and selection

#### Screening of B-cell epitopes

B-cell epitopes are fundamental components in multi-epitope vaccine design, as they trigger adaptive immune responses^32^. The ABCpred platform was employed to predict linear B lymphocyte (LBL) epitopes in protein sequences using an artificial neural network^33^. With the epitope length set to 16, only epitopes with a score exceeding 0.8 were selected, and the remaining parameters were set as default. After submitting the protein sequences to the server, Next, the VaxiJen 2.0, ToxinPred, and AllerTOP v2.1 Platforms were employed to evaluate their antigenicity, toxicity, and allergenicity, respectively^34–36^. For antigenicity assessment via VaxiJen 2.0, the organism was specified as bacteria, and thresholds below 0.4, labeled as "probable non-antigens," were excluded; this server achieves an accuracy of 89%^34^.

#### Screening of T-cell epitopes

The identification of potential MHC-binding sites involved the indirect prediction of T-cell epitopes based on the structural data derived from the target proteins. The IEDB MHC I Binding server was employed to identify CTL epitopes of *T. pallidum* adhesin proteins^37^. The "27 Allele Panel" was selected for MHC alleles, with the remaining parameters set as default. Epitopes with a percentile of less than 0.5 were selected. Concurrently, the MHC I Immunogenicity Platform was utilized to assess the immunogenicity of these epitopes^38^. Subsequently, the HTL epitopes were identified using the IEDB MHC II binding server^39^. The default prediction method was NetMHCpan 4.1 EL, with the epitope length set to 15, and epitopes with a percentile rank of less than 0.5 were selected. To assess the capacity of these epitopes to induce interferon-gamma (IFN-γ), the IFNepitope online server was employed^40^. It is anticipated that these epitopes play a crucial role in eliciting responses from CTL and HTL, thereby activating adaptive immunity. To evaluate the conservation of candidate epitopes across different strains, we adopted the analytical method established by Roohparvar Basmenj et al., with the detailed results provided in Supplementary Materials Table S2^41^.

#### Population coverage analysis

Allele frequencies and distribution rates of different MHC alleles vary significantly across distinct geographical regions and ethnic populations. Therefore, to evaluate the potential population coverage of the designed vaccine, this study employed the Population Coverage Calculation Tool from the IEDB^42^.

### T-cell epitope-MHC molecular interactions

T cell-mediated cellular immune responses are a fundamental defense mechanism against tumors and infections, with specificity determined by the recognition of peptide-MHC complexes (pMHC) and TCR. To forecast immunogenicity, molecular docking techniques were used to model the interactions of T-cell epitopes with MHC alleles. The three-dimensional (3D) structure conformation of T-cell epitopes was generated using the PEP-FOLD3 web server and subsequently saved as PDB files^43^. Structures of canonical MHC alleles were sourced from the RCSB Protein Data Bank (RCSB PDB) including HLA-A*68:01 (PDB: 6PBH), HLA-B*35:01 (PDB: 4LNR), HLA-A*03:01 (PDB: 7L1B), HLA-A*02:03 (PDB: 3OX8), HLA-B*51:01 (PDB: 4MJI), HLA-B*58:01 (PDB: 7WZZ), HLA-B*58:01 (PDB: 5IM7) and HLA-DRB1*04:01 (PDB: 5JLZ)^44^. Docking of epitopes to MHC was conducted on the HawkDock platform, and the binding free energy for each complex was recorded^45^. The resulting poses were visualized through PyMOL, where hydrogen bonding interactions were detected, and the relevant amino acid residues were annotated.

### Vaccine design and analysis

#### Coupling of epitopes with molecular adjuvants

The screened epitopes were connected using linkers to facilitate proper protein folding and processing. Commonly used linkers include GPGPG, AAY, and KK. The GPGPG linker can disrupt the formation of linked epitopes, thereby helping to restore the immunogenicity of individual epitopes. The AAY linker serves as a proteasomal cleavage site in mammalian cells, facilitating the formation of native epitopes to enhance epitope presentation and vaccine immunogenicity. The KK linker provides flexibility and spatial separation for the construction of functional domains or epitopes in proteins^46^. However, the use of inappropriate linkers may adversely affect the functionality of fusion proteins, leading to reduced expression levels, protein misfolding, or impaired bioactivity^47^. During the construction of fusion proteins, β-defensin was incorporated as an immunological adjuvant to augment vaccine immunogenicity^48^. The rigid peptide linker EAAAK is commonly employed to conjugate immune adjuvants to epitopes. It can effectively maintain a fixed distance between functional domains. This strategy not only improves the structural stability of the fusion protein but also minimizes interactions between the adjuvant and adjacent protein regions^49^. Additionally, a dendritic cell (DC) signal peptide was fused to the C-terminus of the epitope sequence to augment the efficiency of antigen presentation^50^.

#### Analysis of vaccine properties

Investigating the impact of epitope order on vaccine structure, we randomized the MEVTP epitope sequence to generate ten vaccine constructs with distinct epitope arrangements. Antigenicity, solubility, molecular aggregation propensity, and intrinsic disorder propensity were assessed using the VaxiJen 2.0, Protein-Sol, Aggrescan, and IUPred servers^51–53^. The optimal structure was selected for further analysis. The physicochemical properties were assessed using the ProtParam module within the ExPASy computational platform^54^. This analysis focused on key descriptors, among others, including molecular mass, theoretical isoelectric point (pI), and grand average hydropathicity (GRAVY). According to the references, proteins with an instability index of less than 40 are classified as stable proteins. Transmembrane helix structures and signal peptides were predicted using the DeepTMHMM and SignalP 6.0 servers^55^. These predictive analyses provide foundational data to guide subsequent experimental studies.

#### Analysis of secondary structures

The two-dimensional (2D) of the vaccine design was forecasted utilizing the PSIPRED and SOPMA servers^56,57^. A protein’s secondary structure, relating to the configuration and folding of its polypeptide chain, primarily comprises α-helices, β-sheets, β-turns, and random coils. Analyzing the percentages of secondary structures allows for the determination of the vaccine’s conformation, as these percentages are critical for predicting the 3D structure of proteins^58^.

#### Modeling and refinement of tertiary structures

The 3D structure of the tandem epitope peptide was modeled via AlphaFold 3, an artificial intelligence-based tool that predicts protein structures based on amino acid sequences^59,60^. Subsequently, the predicted structure was refined using GalaxyRefine, which excels in enhancing local structural quality^61^. Finally, the optimized model was validated on the ProSA-web and SAVES v6.1 servers. In ProSA-web, the Z-score is an indicator of the model’s overall quality. The residue score plot, which is created by plotting energy values against amino acid sequence positions (i), illustrates the local quality of the model^62^. In this context, negative values signify structurally sound segments^62,63^. SAVES v6.1 incorporates the PROCHECK and ERRAT modules^64^. The Ramachandran plot produced by PROCHECK assesses the stereochemical integrity of a protein structure by examining the geometry of individual residues along with the overall fold.

#### Molecular docking

The effectiveness of a multi-epitope vaccine in generating strong immune responses in the host largely relies on the interactions between the proteins of the vaccine and the immune cells of the host^65^. TLR2 and TLR4 have been established as pivotal mediators of *T. pallidum*-triggered inflammatory responses^66,67^. The 3D configurations of TLR2 (PDB ID: 3A7B) and TLR4 (PDB ID: 2Z62) were downloaded from the RCSB PDB. The HawkDock computational platform was utilized to conduct molecular docking, with the vaccines designated as the receptor. Furthermore, the free energy of the complexes was estimated. At last, the interactions at the residue level within the vaccine complex were confirmed and visualized through the PDBsum database^68^.

#### Molecular dynamics simulation assessment

Molecular dynamics (MD) simulations were performed to explore the physical motions and interactions within the docked complex, thereby confirming the stability and binding affinity between the candidate vaccine and the TLR2/4 docking structure. The simulations utilized GROMACS 2022.3, maintaining controlled conditions of 300 K and 1 bar, with the Amber99sb-ildn force field and the TIP3P water model, while being neutralized with Na⁺ ions^69,70^. The system went through energy minimization via the steepest descent approach, succeeded by a sequential equilibration in both the isothermal-isovolumetric ensemble and the isothermal-isobaric ensemble, culminating in a 100 ns free MD simulation. The analysis of the trajectory calculated various metrics, including the Radius of Gyration (Rg), Root Mean Square Fluctuation (RMSF), and Solvent Accessible Surface Area (SASA), Root Mean Square Deviation (RMSD), among others.

Additionally, the post-docking results were submitted to the iMODS website for analysis^71^. The platform was applied to investigate the collective movements of proteins and nucleic acids through Normal Mode Analysis within internal coordinates, specifically in torsional space. To conduct this analysis, atomic coordinates in PDB format must be submitted, with backbone atoms N, CA, and C being essential for defining dihedral angles.

#### Prediction of structural B-cell epitopes

Antibodies need to interact with antigenic epitopes to eliminate pathogens. Therefore, predicting conformational epitopes is of crucial importance. ElliPro identifies epitopes using the three-dimensional structure of protein antigens; residues protruding from the protein surface are more likely to bind to antibodies, and these protruding residues can be identified by treating the protein as an ellipsoid^72^. On this basis, the present study employed ElliPro for the prediction of both linear and discontinuous B-cell epitopes. Selection criteria were defined with a minimum score of 0.5. Structural epitopes are likely to deviate following any modeling optimization. Therefore, conformational epitope prediction was based on the final snapshot obtained from 100 ns MD simulations, which can yield a more reliable conformation.

#### Codon adaptation and plasmid construction

Codon optimization is a critical strategy for aligning the codon usage of the gene with that of the expression host, which is vital for enhancing heterologous protein expression. In this research, the Optimizer server was utilized for codon optimization^73^. The Codon Adaptation Index (CAI), indicative of how well the optimized codons align with the host organism’s codon preferences, correlates with strong protein expression when the CAI value surpasses 0.8, and the Guanine-cytosine (GC) composition is between 30 and 70%^74^. Subsequently, the optimized sequence was integrated into the prokaryotic expression vector using SnapGene software, with the pET-28a( +) plasmid vector selected and restriction enzymes *XhoI* and *BamHI* employed. Finally, the target protein was expressed in *Escherichia coli (E. coli)* using the constructed recombinant plasmid.

#### Construction and prediction of vaccine mRNA

To enhance mRNA stability and translational efficiency, this study adopted the design strategy proposed by Suneesh et al*.*^75^. A Cap 1 structure was incorporated at the 5′ terminus to prevent degradation, followed by the introduction of the 5′UTR from the human β-globin gene (UCSC ID: ENST00000335295.4) to optimize translation efficiency^76^. Additionally, a Kozak consensus sequence and a signal peptide were inserted. The 3′UTR was adopted from the mRNA-1273 COVID-19 vaccine (sourced from a GitHub repository), while a segmented poly(A) tail was appended to augment stability^77^. Finally, RNAfold predicted the secondary structure using dynamic programming algorithms with thermodynamic parameter constraints for chemically modified nucleotides, calculating the minimum free energy (MFE) conformation^78^.

#### Immune simulation

The C-ImmSim computational platform can be employed by researchers to computationally forecast the immune responses triggered by multi-epitope vaccines in organisms^79^. This method is essential for evaluating the anticipated efficacy of vaccines. C-ImmSim mainly assesses the reliability of vaccine designs by forecasting the generation of antibodies, interferons, and cytokines after interactions between the vaccine and the host^80^. The server simulates immune responses across three mammalian anatomical compartments: the thymus, bone marrow, and lymphoid organs^79^. For the in silico simulations, three distinct vaccine doses were tested. The simulation process was established with a cumulative count of 1050 steps, with time intervals set at 1, 84, and 170. Each of these periods represents a genuine duration of 8 h, with the initial time segment indicating the beginning point for vaccination. All additional parameters were maintained at their standard configurations. The simulations conducted under these parameters were meticulously designed to accurately reflect the dynamics of the immune response associated with the vaccine.

### Protein expression

The MEVTP gene was synthesized by Suzhou Junji Gene Technology Co., Ltd. and subsequently inserted into the pET-28a( +) vector, yielding the recombinant plasmid pET-28a( +)-MEVTP, which enables the expression of a protein containing a 6 × His tag. The plasmid was introduced into *E. coli* BL21(DE3). The recombinant *E. coli* strain was inoculated onto LB solid plates containing kanamycin. A colony was selected and inoculated into LB liquid medium with kanamycin, followed by an overnight culture at 37 ℃ and 220 rpm. The bacterial culture was then scaled up until the OD600 reached 0.6. Isopropyl-β-D-thiogalactoside (IPTG) was added to a final concentration of 0.2 Millimolar to induce protein expression at 16 ℃ and 160 rpm for 12 h. After cell disruption via sonication, the pellet was collected to obtain the target protein, which was subsequently purified using a nickel affinity chromatography column. Elution was performed using a gradient of imidazole from 20 to 500 Millimolar. The results were analyzed by sodium dodecyl sulfate–polyacrylamide gel electrophoresis (SDS-PAGE). Western blot analysis was performed using an anti-His-tag antibody to confirm the identity of the target protein.

## Results

### Characterization of *T. pallidum* adhesins

The predicted properties of *T. pallidum* adhesin proteins are summarized in Table 1. Certain adhesin proteins exhibit favorable antigenicity, satisfactory solubility, and non-homology. Among these proteins, Tp0155 and Tp0750 have antigenicity scores below the VaxiJen threshold of 0.40. However, since antigenicity at the epitope level is the most relevant factor for vaccine development, the overall advantageous properties of these proteins still facilitate the development of multi-epitope vaccines. To further clarify the homologous clustering relationships of these candidate proteins, we constructed their phylogenetic trees, and the relevant results are provided in Supplementary Fig. 1.

**Table 1:** Adhesin proteins prediction results.

Protein	Antigenicity	Solubility	Molecular weight (KDa)	Theoretical pI	Instability index	Homology
Tp0136	1.1954	0.290	56.226	8.42	45.48	NO
Tp0155	0.3818	0.267	58.819	9.82	42.83	NO
Tp0435	0.5121	0.799	16.467	8.88	33.11	NO
Tp0483	0.5903	0.335	42.106	9.33	34.02	NO
Tp0750	0.3936	0.404	25.940	9.08	51.27	NO
Tp0751	0.5794	0.695	25.770	7.30	44.56	NO
Tp0954	0.4312	0.515	54.661	8.83	39.84	NO

### Epitope profiling and prioritization

#### B-cell epitopes selection

Epitopes with ABCpred scores greater than 0.8 were preferentially selected, provided they were non-toxic and non-allergenic. A complete list is provided in Table 2.

**Table 2:** Results of B-cell epitopes.

Protein	Epitope	Start–end	ABCpred score	Antigenicity	Allergenicity	Toxicity
Tp0136	TSSTQRPDLYAAVGES	462–477	0.86	0.9079	NO	NO
Tp0155	TGRSTGPHLHFTIYKN	342–357	0.80	0.9121	NO	NO
Tp0435	VSSEQSKAPHEKELYE	110–125	0.80	0.7419	NO	NO
Tp0483	LKTGSYTLRAITPRNI	183–198	0.80	0.8210	NO	NO
Tp0750	DLEHDAPLTSKYRGKQ	141–156	0.88	0.6173	NO	NO
Tp0751	SGSSTTTDPRSHGNAP	70–85	0.96	1.5671	NO	NO
Tp0954	TGSNSARESERAQLLK	25–40	0.85	1.2859	NO	NO

#### T-cell epitopes selection

Using the IEDB, we predicted the MHC-I and MHC-II epitopes of the adhesin protein. After evaluating allergenicity, toxicity, antigenicity, potential for IFN-γ induction, and immunogenicity, we selected 11 CTL and 4 HTL epitopes for vaccine construction, as represented in Tables 3 and 4.

**Table 3:** Results of CTL epitopes.

Protein	Epitope	Start–end	Allele	Vaxijen score	Allergenicity	Toxicity	Immunogenicity
Tp0136	EQYRGTVGR	416–424	HLA-A*33:01, HLA-A*68:01,	1.0605	NO	NO	0.17506
Tp0155	GPHLHFTIY	345–353	HLA-B*35:01, HLA-B*53:01	1.6476	NO	NO	0.25156
	ALLLFVTLL	23–31	HLA-A*02:01	0.8249	NO	NO	0.16438
Tp0435	YMGAPGAGK	132–140	HLA-A*03:01	0.8154	NO	NO	0.12429
Tp0483	YLYELYPRI	292–300	HLA-A*02:01, HLA-A*02:03, HLA-A*02:06, HLA-A*32:01	1.7630	NO	NO	0.10615
	KADEAGAYV	151–159	HLA-A*02:06	0.9413	NO	NO	0.20881
	KEQEARISW	31–39	HLA-B*40:01, HLA-A*32:01	1.5428	NO	NO	0.16563
Tp0750	VPVDIFLMI	27–35	HLA-B*51:01, HLA-B*53:01	1.7758	NO	NO	0.16356
	ALCTFLIHL	10–18	HLA-A*02:01, HLA-A*02:06, HLA-A*02:03	1.7072	NO	NO	0.25634
Tp0751	VAAWALYIF	14–22	HLA-B*58:01	2.1229	NO	NO	0.33789
Tp0954	YSFYLAFFY	422–430	HLA-A*30:02, HLA-A*01:01, HLA-A*26:01, HLA-B*58:01	2.4674	NO	NO	0.22751

**Table 4:** Results of HTL epitopes.

Protein	Epitope	Start–end	Allele	Antigenicity	Allergenicity	Toxicity	IFN-g scores
Tp0136	QKIYVVEKNGGGNGV	428–442	HLA-DRB1*04:01, HLA-DRB1*08:02, HLA-DRB1*11:01	1.5050	NO	NO	0.057082655
Tp0483	VKPSFTGVSLQQTPS	351–365	HLA-DPA1*03:01/DPB1*04:02, HLA-DPA1*02:01/DPB1*01:01	0.9308	NO	NO	0.53606652
Tp0751	IHVRAVEDVARLKIG	201–215	HLA-DPA1*02:01/DPB1*14:01	0.9317	NO	NO	0.13062741
Tp0954	LQLFDTLSPEHRAEK	63–77	HLA-DRB5*01:01	0.8310	NO	NO	0.022496755

#### Population coverage rate

**Fig. 2:**
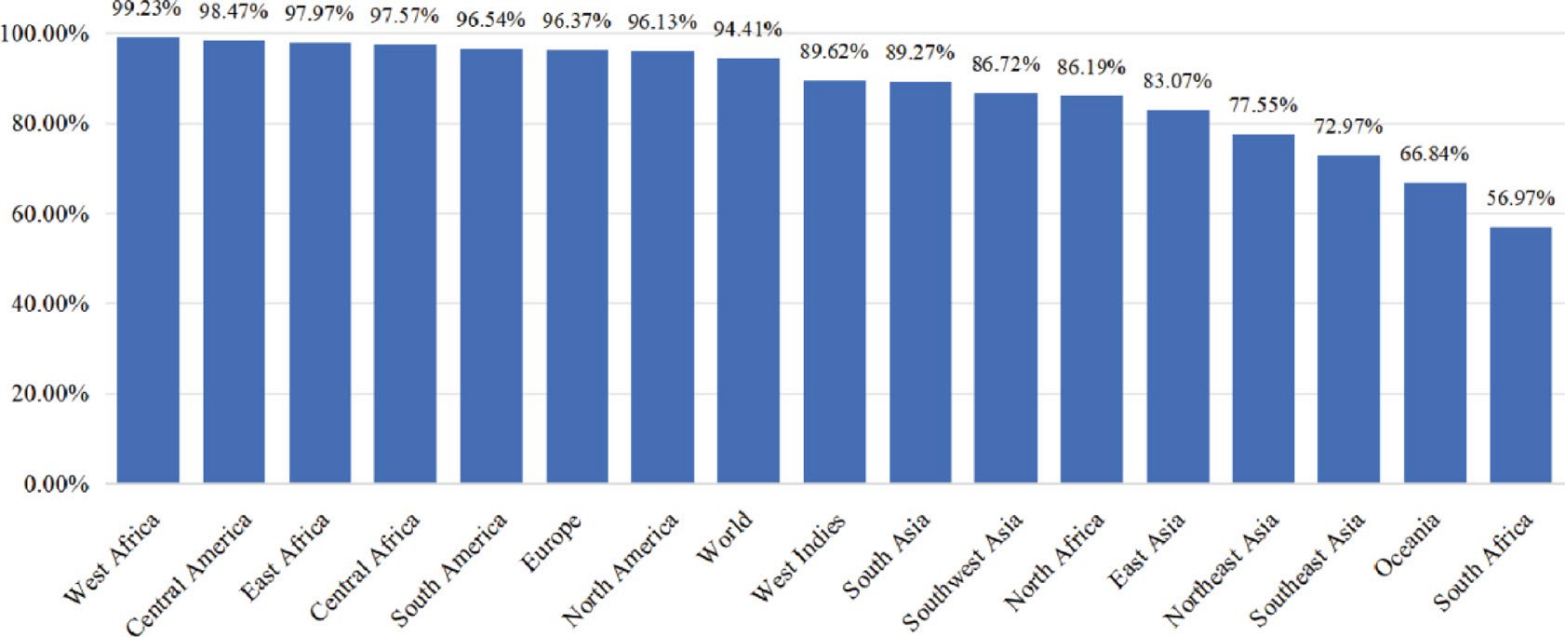
Integrated population coverage of the selected CTL and HTL epitopes. The x-axis denotes country names; the y-axis denotes the integrated population coverage.

We calculated the integrated population coverage based on the selected T-cell epitopes and their corresponding HLA alleles. Population coverage prediction is crucial for vaccine design, as it ensures that the designed vaccine construct can cover the broadest possible global population (Fig. 2).

### T-cell epitope-MHC molecular interactions

**Fig. 3:**
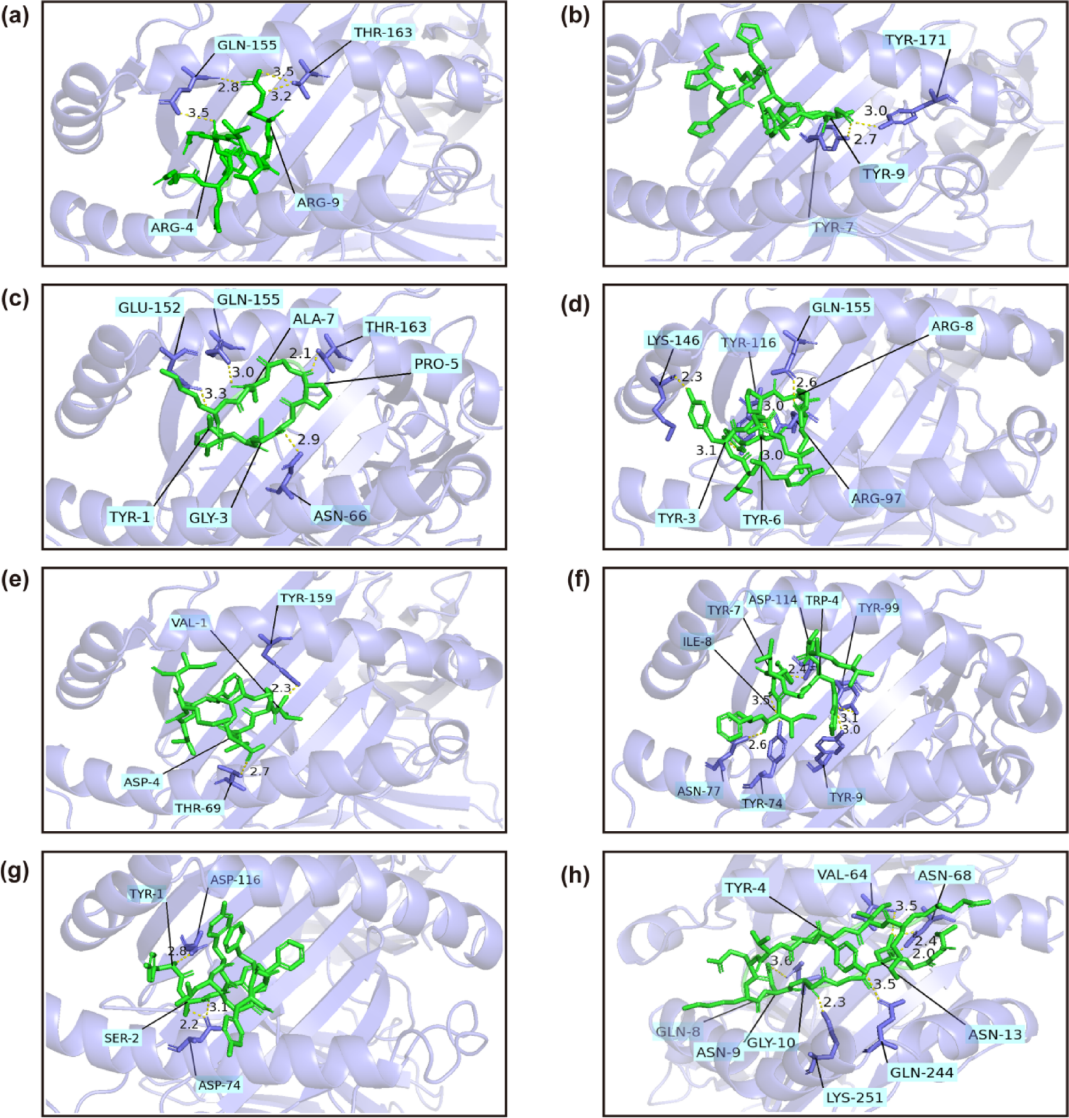
Assessment of peptide-HLA interactions. (**a**) Molecular docking of Tp0136 EQYRGTVGR with HLA-A*68:01 (PDB: 6PBH) yielded a ΔG of − 32.0 kcal/mol. (**b**) Molecular docking of Tp0155 GPHLHFTIY with HLA-B*35:01 (PDB: 4LNR) resulted in a ΔG of − 31.0 kcal/mol. (**c**) Molecular docking of Tp0435 YMGAPGAGK with HLA-A*03:01 (PDB: 7L1B) produced a ΔG of − 42.8 kcal/mol. (**d**) Molecular docking of Tp0483 YLYELYPRI with HLA-A*02:03 (PDB: 3OX8) revealed a ΔG of − 44.37 kcal/mol. (**e**) Molecular docking of Tp0750 VPVDIFLMI with HLA-B*51:01 (PDB: 4MJI) showed a ΔG of − 21.17 kcal/mol. (**f**) Molecular docking of Tp0751 VAAWALYIF with HLA-B*58:01 (PDB: 7WZZ) indicated a ΔG of − 46.77 kcal/mol. (**g**) Molecular docking of Tp0954 YSFYLAFFY with HLA-B*58:01 (PDB: 5IM7) resulted in a ΔG of -53.36 kcal/mol. (**h**) Molecular docking of Tp0136 QKIYVVEKNGGGNGV with HLA-DRB1*04:01 (PDB: 5JLZ) demonstrated a ΔG of -34.59 kcal/mol.

The complex formed by T-cell epitopes binding to MHC molecules activates antigen-specific T cells. These cells subsequently differentiate into effector T cells, which mediate immediate cellular immune responses and establish memory T-cell subsets that confer long-term immune protection. In this study, molecular docking experiments were conducted between candidate peptides and their corresponding human leukocyte antigen (HLA) molecules to evaluate binding affinity. All selected epitopes are located within the binding grooves of their respective alleles. The results are illustrated in Fig. 3. This data provides critical insights into peptide-HLA interactions and establishes a rational basis for epitope screening in vaccine design.

### Vaccine construction and validation

#### Multi-epitope vaccine construction

Epitopes were interconnected utilizing specific linkers. The ultimate vaccine construct consists of β-defensin, B-cell epitopes, CTL epitopes, HTL epitopes, and a DC signal peptide. The impact of epitope ranking on vaccine architecture is shown in Supplementary Material Table S3. The aggregation propensity was predicted using the Aggrescan server, with lower values deemed favorable. The intrinsic disorder propensity was assessed via the IUPred server, with scoring between 0 and 1 inclusively; higher scores indicate a greater likelihood of residues belonging to disordered regions, while lower disorder is advantageous for vaccine design. Sequence identity between protein pairs was evaluated with the SIM-Alignment Tool, which calculates the percentage of identical amino acid residues.

The differences in the ranking results are not significant, exhibiting minimal variation. Consequently, MEVTP-1, MEVTP-3, MEVTP-8, and MEVTP-10 were prioritized for immune simulation (see Supplementary Materials Fig. S2). Following a comprehensive analysis, MEVTP-10 was selected for further investigation. See Table 5. The predicted properties of this vaccine construct are as follows: an antigenicity score of 0.9425, indicating its capacity for specific binding with induced antibodies; a pI of 9.76, suggesting an alkaline characteristic; an instability index of 37.59, categorizing it as stable; and an aliphatic index of 68.45, which reinforces its robust thermostability. Additionally, the molecule’s GRAVY value was − 0.495, reflecting hydrophilic characteristics. The lack of allergenicity, transmembrane helices, and signal peptides in this protein reduces the potential risks of adverse reactions. With a molecular weight of 44 kDa and a solubility score of 0.527, MEVTP-10 demonstrates high feasibility for downstream purification and expression processes, thereby providing robust support for subsequent vaccine development and application.

**Table 5:** Properties of multi-epitope vaccines.

Vaccine	MEVTP-10
Antigenicity	0.9425
Instability index	37.59
Solubility	0.527
Molecular weight(KDa)	44 kDa
Theoretical pI	9.76
Aliphatic index	68.45
GRAVY	− 0.495
Signal peptide	0
Transmembrane topology	0
Allergenicity	No

#### Analysis of secondary and tertiary structures

**Fig. 4:**
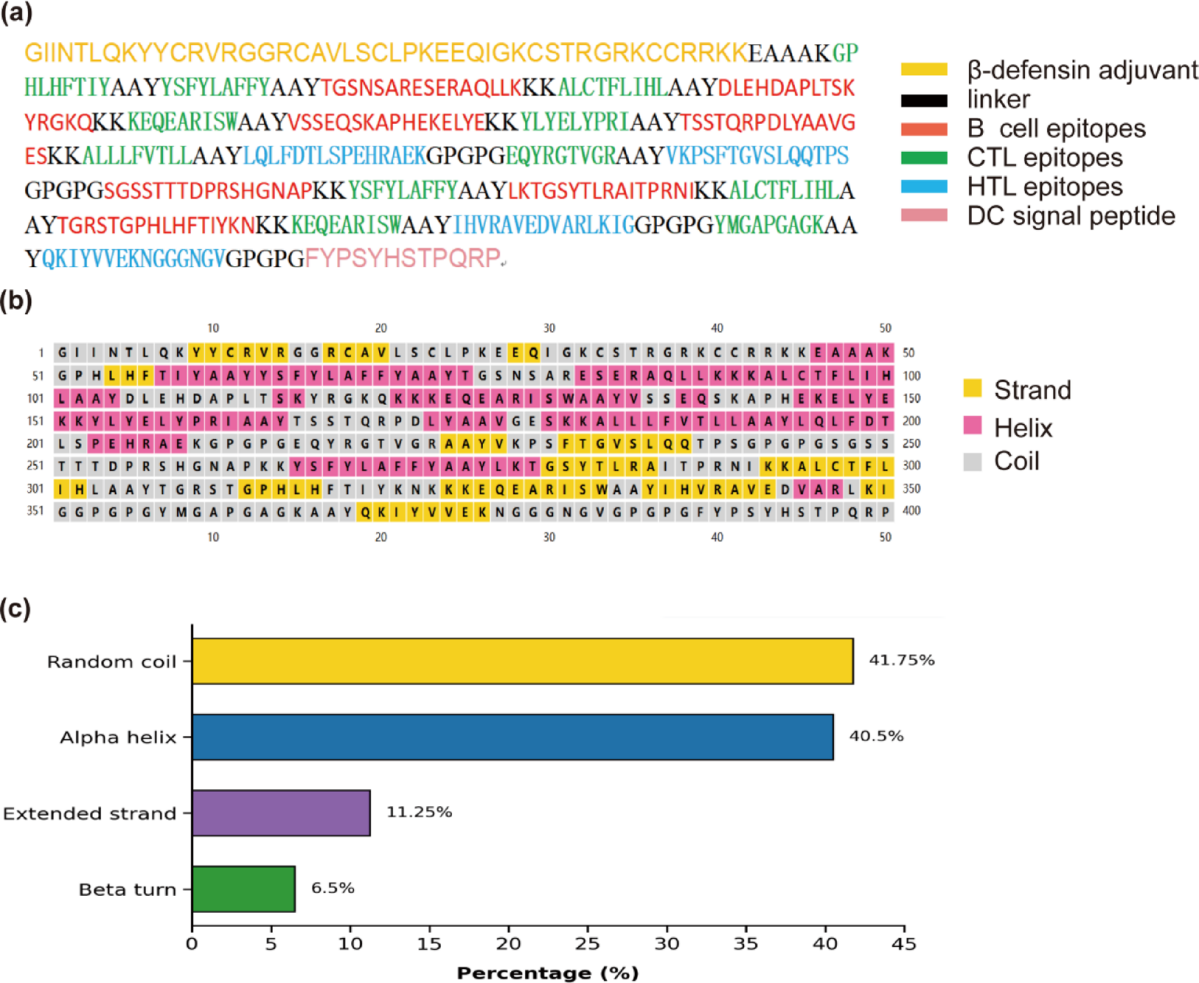
Protein secondary structure features and epitope distribution. (**a**) Connectivity of epitopes within the protein structure. (**b**) The amino acid sequence of the protein is color-coded to represent its 2D structure. (**c**) This section presents the percentage composition of various 2D structure types within the protein.

**Fig. 5:**
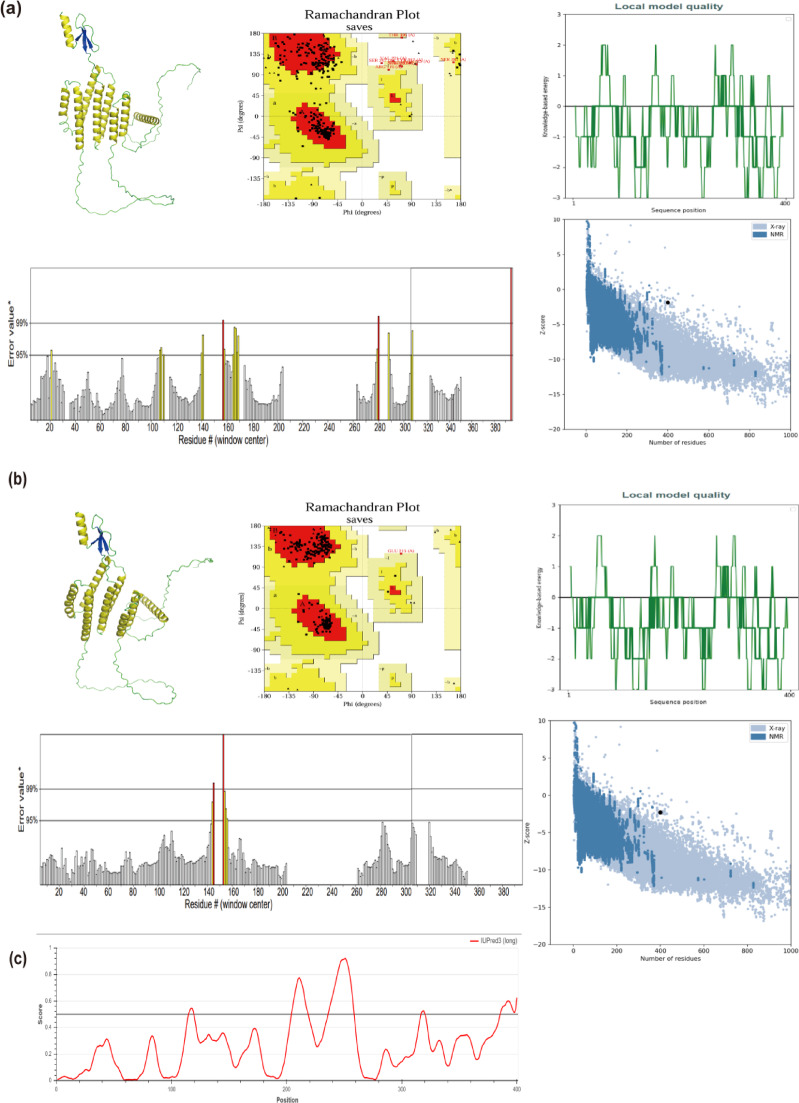
Tertiary structure prediction and optimization. (**a**) The 3D structure, PROCHECK Ramachandran plot, Z-score, Local model quality, and ERRAT overall quality of the AlphaFold model. (**b**) Shows the corresponding assessments for the GalaxyreWEB model. (**c**) Disorder prediction plot.

As shown in Fig. 4, the composition of the 2D structure was: 40.50% alpha-helix, 11.25% extended strand, 6.50% beta-turn, and 41.75% random coil. The 3D structure analysis indicates that the structural validation of the AlphaFold model showed significant improvements following refinement. For the AlphaFold-predicted model (Fig. 5a), the Ramachandran plot analysis revealed that 84.6% of residues were in core regions, 12.7% in allowed regions, 2.4% in generously allowed regions, and 0.3% in disallowed regions, accompanied by a ProSA-web Z-score of -1.87 and an ERRAT value of 92.017. After refinement using the GalaxyWEB server, the GalaxyRefine-optimized model (Fig. 5b) exhibited markedly enhanced stereochemical quality: 98.2% of residues occupied core regions, 1.5% in allowed regions, 0.3% in generously allowed regions, and no disallowed residues (0.0%), with a ProSA-web Z-score of − 2.28 and an ERRAT value of 97.778. Well-determined, high-resolution structures typically exhibit values of ≥ 95%. The Ramachandran plot indicates that a proportion of amino acid residues exceeding 90% in the core and allowed regions suggests a plausible conformation of the vaccine’s 3D model. We supplemented a disorder prediction plot alongside the 3D structure to clarify the inherent flexibility of the linker regions.

#### Molecular docking analysis

**Fig. 6:**
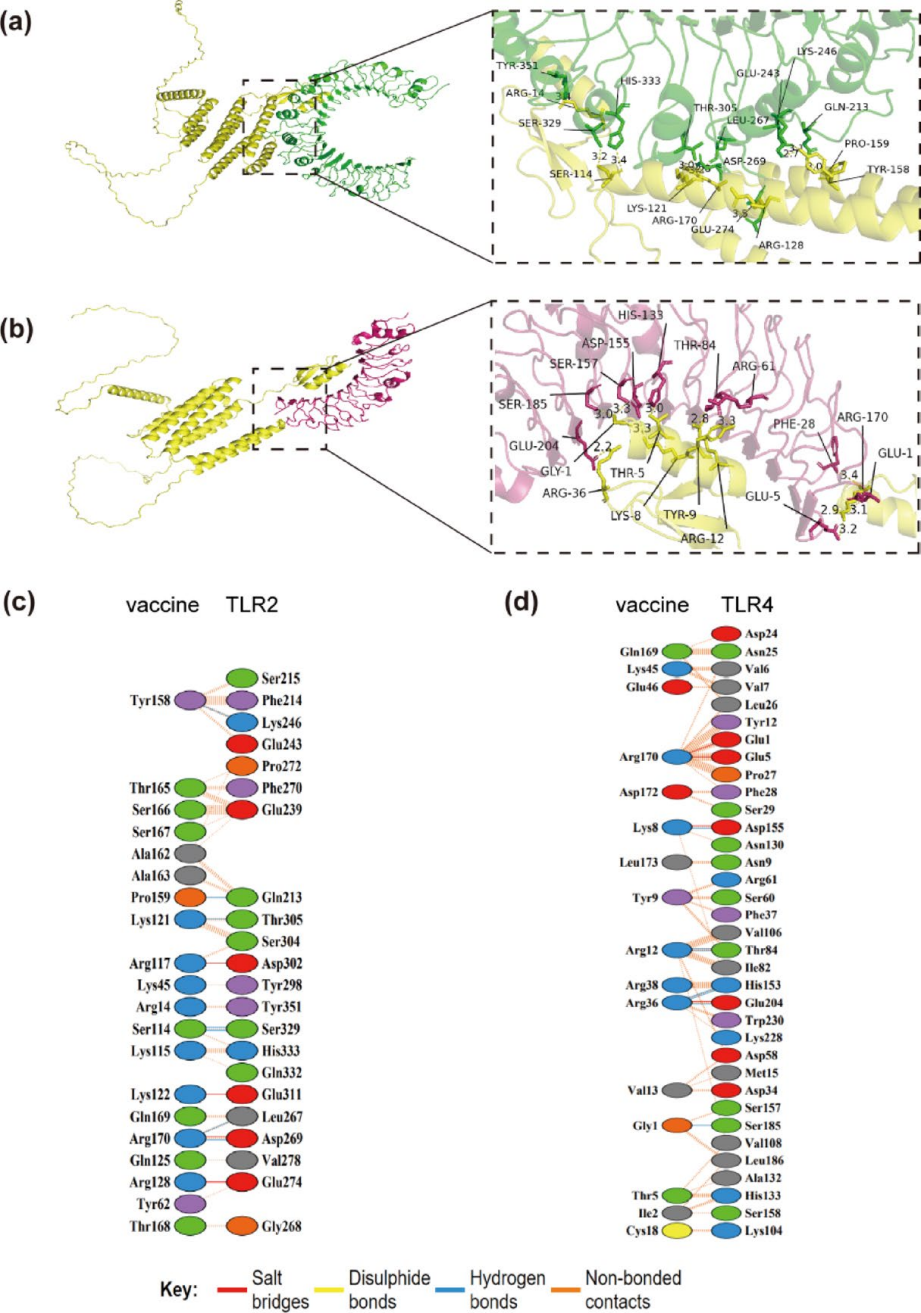
The structure for molecular docking was created with the HawkDock server, and the interactions were examined using the PDBsum server. (**a**, **b**) The binding of the vaccine to TLR2/4 complexes was visualized through PyMOL rendering. (**c**, **d**) A schematic model of the inter-chain interactions between the vaccine and TLR2/4 is presented.

Molecular docking studies revealed significant interactions between TLRs and the fusion protein (Fig. 6). Analysis using the HawkDock server indicated that TLR2 exhibited a binding energy of − 80.89 kcal/mol, a HawkDock score of − 5446.15, and formed seven hydrogen bonds along with four salt bridges, as determined by the PDBsum server. In contrast, TLR4 demonstrated a binding energy of − 69.85 kcal/mol, a HawkDock score of − 6424.54, and also formed seven hydrogen bonds and four salt bridges. These findings underscore a robust binding affinity of the fusion protein toward the TLRs. See Table 6 for details.

**Table 6:** Molecular docking data. Binding free energy is composed of van der Waals energy (ΔEvdW), electrostatic energy (ΔEelec), polar solvation energy (ΔGGB), and nonpolar solvation energy (ΔGSA).

Energy Component	MMGBSA(kcal/mol)	
	MEVTP10-TLR2	MEVTP10-TLR4
ΔEVDW	− 109.41	− 118.6
ΔEelec	− 1504.18	− 1842.64
ΔGGB	1548.81	1908.32
ΔGSA	− 16.11	− 16.93
ΔGTOTAL	− 80.89	− 69.85

#### MD simulation analysis

**Fig. 7:**
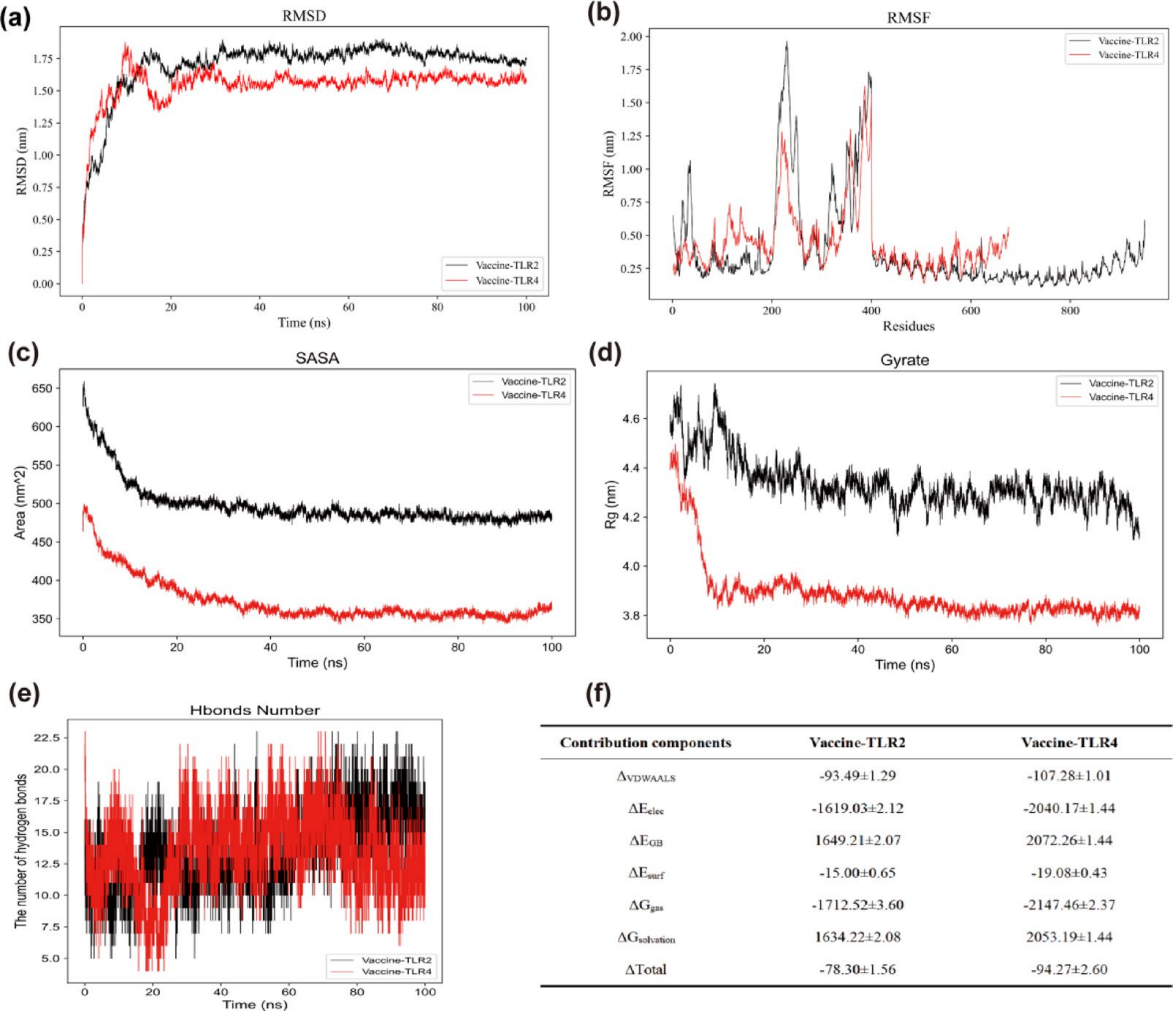
Molecular dynamics simulation analysis. (**a**) RMSD: A metric quantifying structural changes during simulations or accuracy in protein structure prediction. (**b**) RMSF: A measure of residue flexibility in molecular simulations or protein dynamics. (**c**) SASA: A critical metric used to evaluate the exposure of protein molecules in a solution. (**d**) Rg: An indicator of protein compactness and folding state. (**e**) Hbonds Number. (**f**) Binding Free Energy Data.

Comprehensive MD simulations were executed to analyze the Vaccine-TLR2 and Vaccine-TLR4 complexes. As illustrated in Fig. 7, RMSD analysis indicated that the Vaccine-TLR4 complex reached equilibrium around 15 ns, exhibiting lower RMSD values compared to the Vaccine-TLR2 complex, thus indicating enhanced overall conformational stability. RMSF analysis revealed that the average residue fluctuation for Vaccine-TLR2 (0.39 ± 0.35 nm) was slightly lower than that for Vaccine-TLR4 (0.45 ± 0.25 nm). However, the fluctuations in Vaccine-TLR4 were more evenly distributed across residues with no significant peaks, suggesting improved overall flexibility control and enhanced local stability. Furthermore, the Vaccine-TLR4 complex demonstrated a significantly smaller SASA compared to the Vaccine-TLR2 complex, contributing to enhanced complex stability. Rg analysis indicated a more compact structure for Vaccine-TLR4 (3.89 ± 0.12 nm) relative to Vaccine-TLR2 (4.34 ± 0.10 nm). Notably, Vaccine-TLR4 achieved structural stability rapidly in the early simulation phase (within ~ 10 ns), signifying a more compact and conformationally focused complex. Hydrogen bond analysis revealed that both complexes maintained a substantial number of hydrogen bonds throughout the simulation, indicative of strong intermolecular interactions and stable binding. Importantly, the total binding free energy was significantly more favorable for Vaccine-TLR4 (− 94.27 ± 2.60 kcal/mol) than for Vaccine-TLR2 (− 78.30 ± 1.56 kcal/mol), confirming its superior binding affinity. In summary, the Vaccine-TLR4 complex exhibited superior performance in several aspects, including overall conformational stability, flexibility control, structural compactness, and binding affinity compared to the Vaccine-TLR2 complex, highlighting its greater structural stability.

**Fig. 8:**
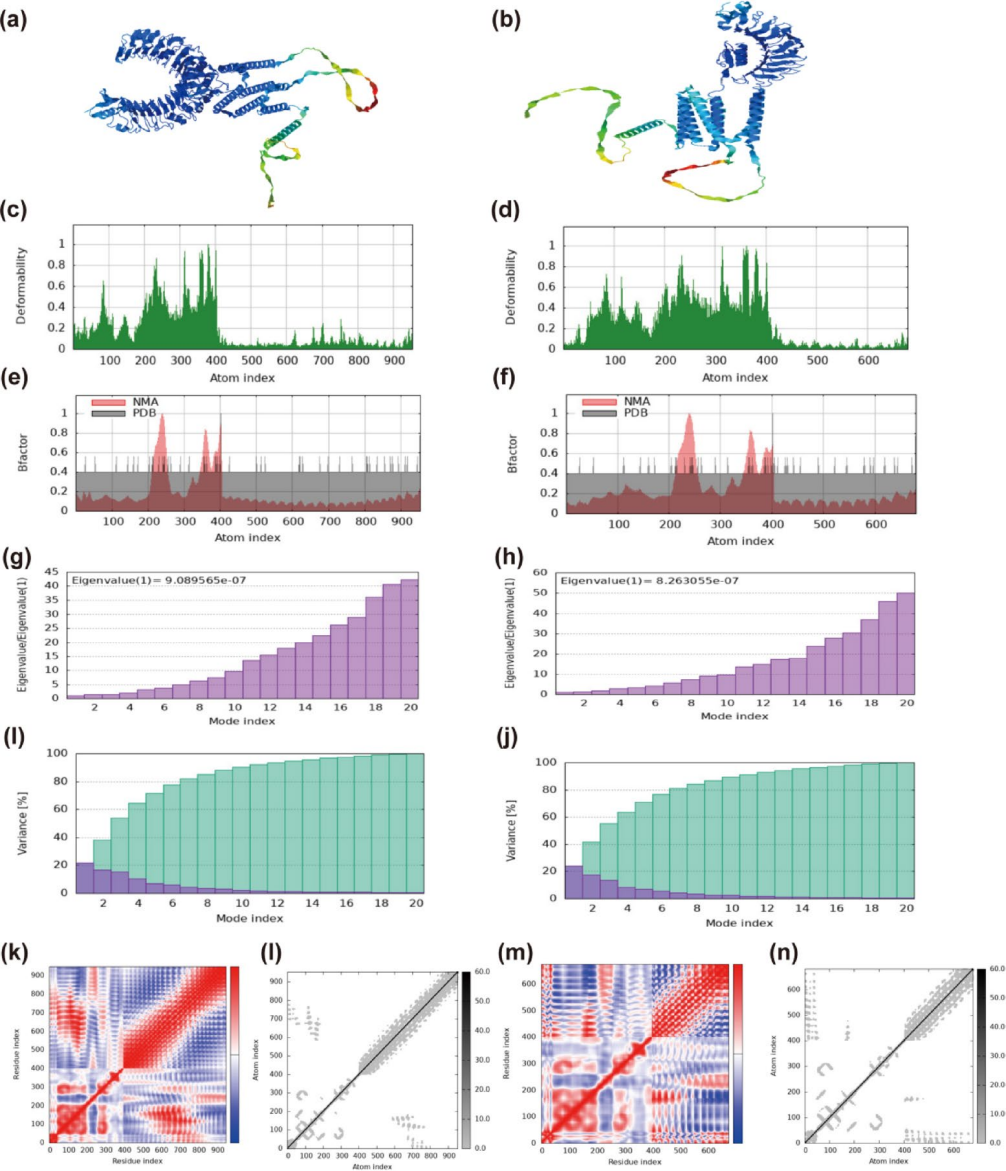
Molecular dynamics simulation results obtained from the iMODS server. (**a**, **b**) Docking of TLR2 and TLR4 with the vaccine, respectively. (**c**, **d**) Backbone mobility per residue. **(e**, **f)** B-factor analysis. **(g-h)** Eigenvalue distribution. (**I**, **j**) Modal variance distribution (individual and cumulative). (**k**, **m**) Residue covariance map. (**l, n**) Elastic network model representation.

The results generated by iMODS are presented in Fig. 8. The mobility plot illustrates the deformability of the backbone per residue, quantifying the molecular deformation capacity. Experimental B-factors were sourced from PDB entries, while calculated NMA B-factors were derived from the product of NMA mobility and 8π^2^. Eigenvalues exhibit direct proportionality to the energy requirements for deformation (TLR2: 9.089565e−07, TLR4: 8.263055e−07), with lower eigenvalues indicating greater ease of deformation. Modal variance exhibits an inverse proportionality to the magnitude of eigenvalues, as illustrated by the individual (purple) and cumulative (green) variance distributions. Residue-pair dynamical couplings are quantified by the covariance matrix using color encoding: red indicates correlation, white signifies no correlation, and blue represents anti-correlation. Within the elastic network model, atomic pairs are represented as harmonic spring potentials, where grayscale intensity denotes stiffness (darker = stiffer springs).

#### Prediction of structural B-cell epitopes

**Fig. 9:**
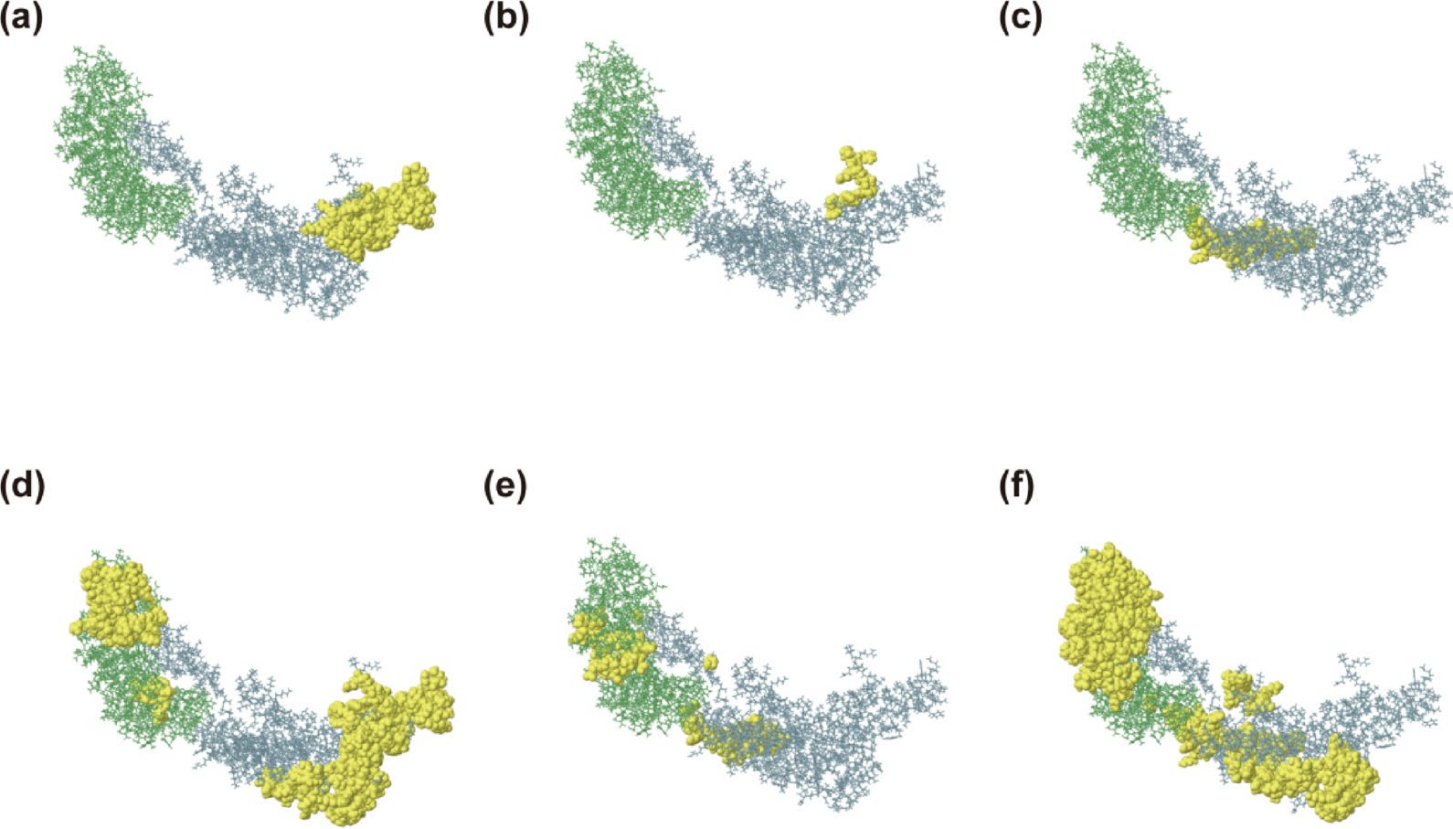
Structural B-cell epitopes identified in the study. (**a**–**c**) Show the linear epitopes. (**d**–**f**) Show the conformational epitopes. The yellow regions represent the epitopes, and the remaining regions correspond to the vaccine–TLR4 complex.

The conformations of the vaccine and TLR4 at the 100 ns time point of the MD simulation were selected for analysis, and a total of fifteen linear B-cell epitopes and six conformational epitopes were identified using the ElliPro server. See Tables S4 and S5 in the supplementary materials. There is a positive correlation between residue scores and solvent accessibility. Figure 9 further presents the top three ranked linear B-cell epitopes and conformational B-cell epitopes.

#### Protein expression

**Fig. 10:**
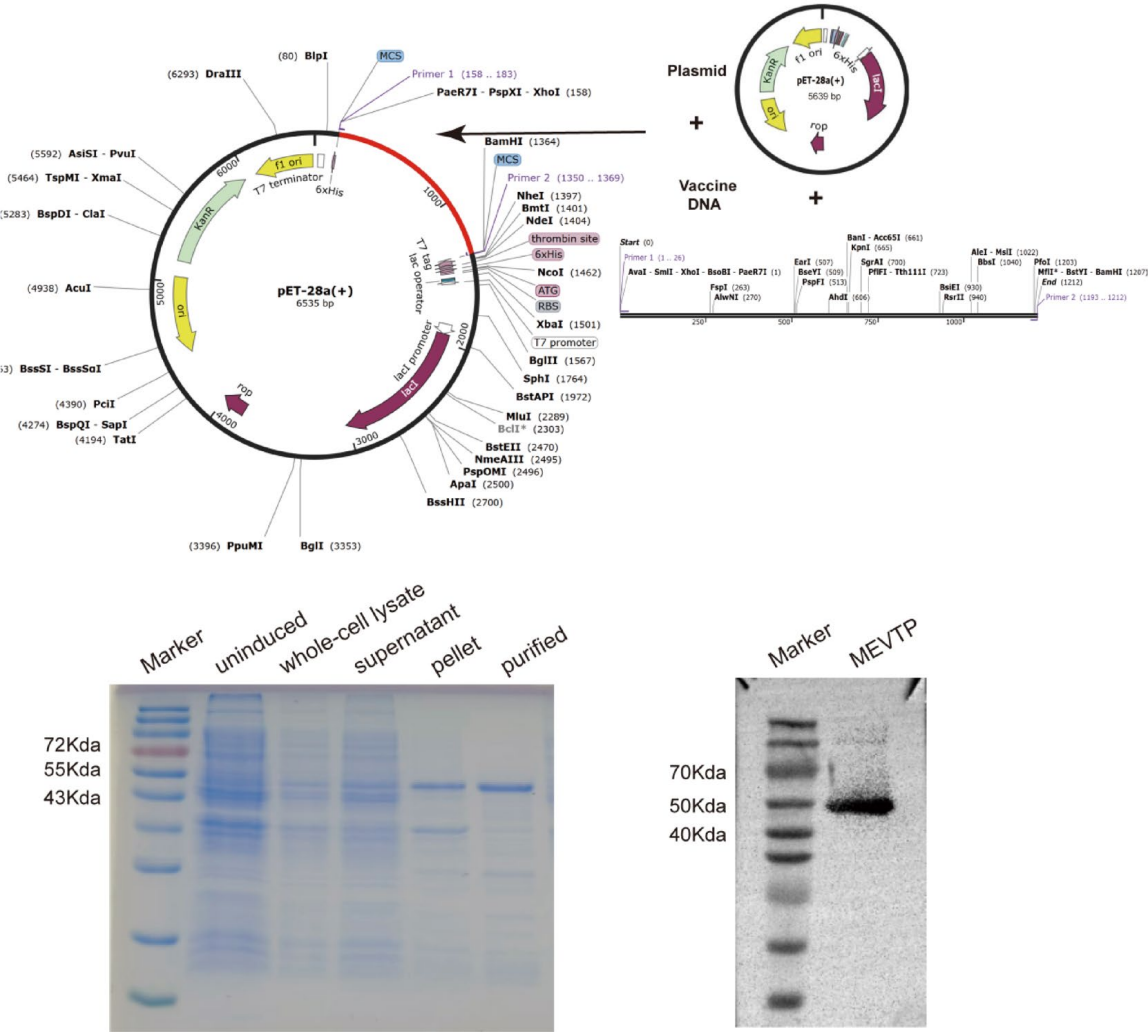
Vaccine construction and protein purification diagram.

The optimized vaccine codon sequence comprised 1,200 nucleotides, demonstrating a GC content of 55.5% and a CAI of 1.0, indicating an optimal alignment with the host’s preferred codon usage. Subsequently, using SnapGene software, molecular cloning was executed to incorporate the vaccine gene into the pET-28a( +) vector, leading to the creation of a recombinant plasmid measuring 6535 base pairs in length. This plasmid was then introduced into the host cells of *E. coli* BL21. Following induction with IPTG, the recombinant protein that was expressed was collected. The desired protein was extracted through Ni^2^⁺ affinity chromatography and subsequently analyzed using SDS-PAGE. Western blot analysis results verified the presence of a protein band at approximately 48 kDa, consistent with the expected molecular weight (see Fig. 10).

#### Prediction of vaccine mRNA

**Fig. 11:**
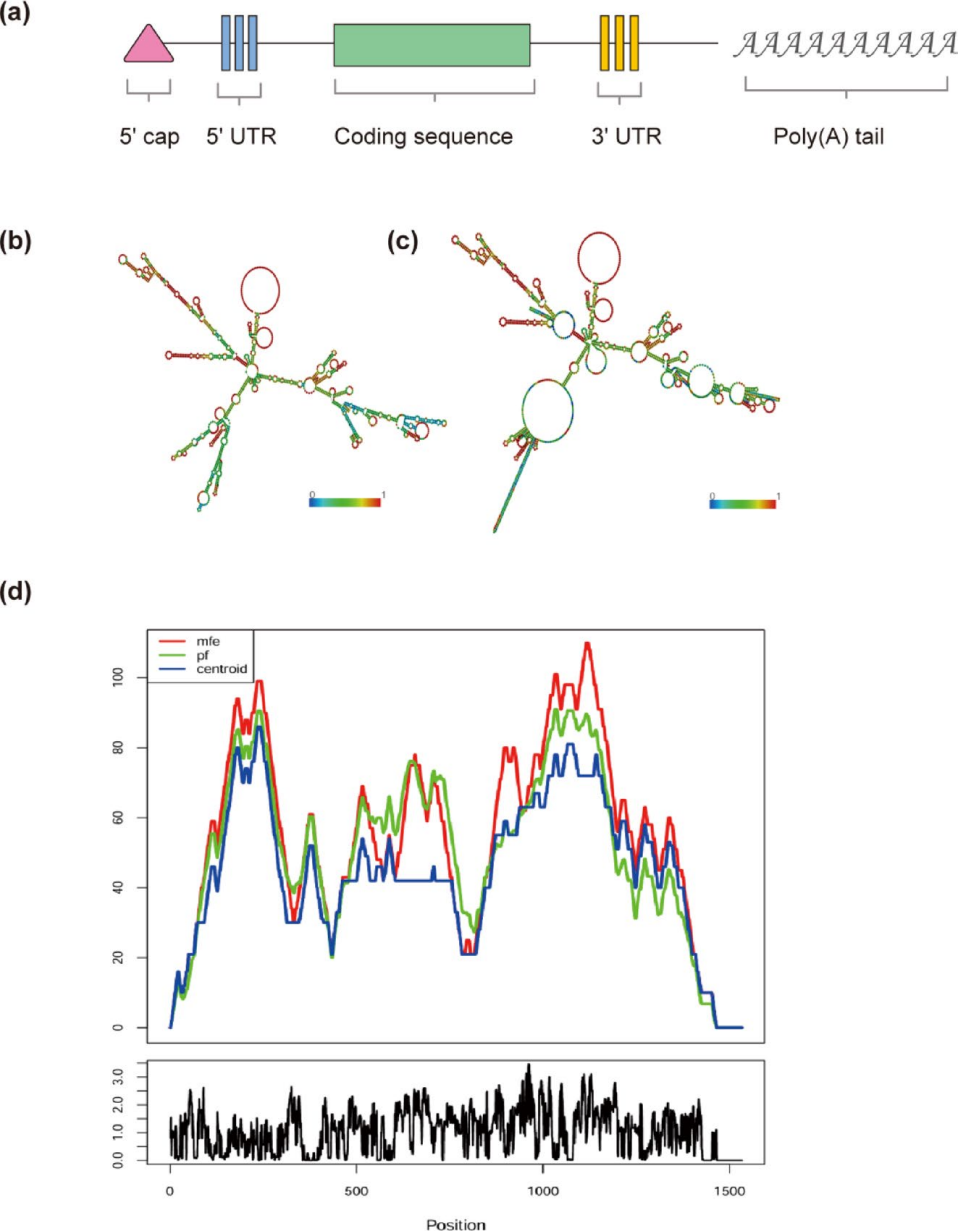
2D structure of vaccine mRNA. (**a**) Depicts the assembly structure of mRNA. (**b**) Shows the MFE structure. (**c**) Represents the centroid 2D structure, where color indicates base-pairing probability. (**d**) Presents a mountain plot that illustrates correlations among the MFE structure, centroid structure, and RNA thermodynamic ensemble, while also indicating sites at each position.

Based on predictions, the optimal secondary structure exhibited an MFE of − 497.80 kcal/mol. The free energy of the thermodynamic ensemble was calculated to be − 521.38 kcal/mol, while the centroid 2D structure demonstrated an MFE of − 343.50 kcal/mol. Lower free energy values indicate greater structural stability (see Fig. 11).

#### Immune simulation

**Fig. 12:**
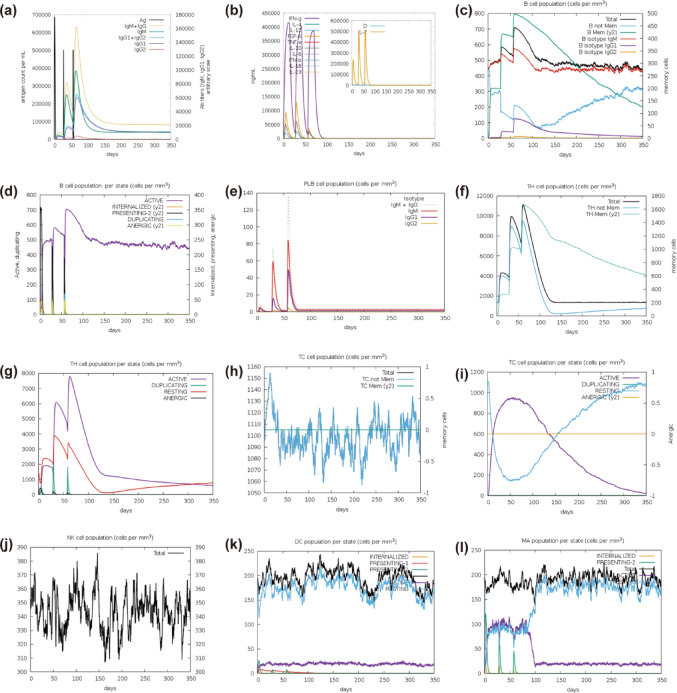
Immune simulation diagram generated from the engineered vaccine. (**a**) Antibody production following antigen exposure. (**b**) Cytokine and interleukin responses. (**c**–**d**) B cell populations (total and class-switched). (**e**) Plasma B cell population. (**f–g**) Helper T cell population. (**h–i**) Cytotoxic T cell population. (**j**) Natural killer cell population. (**k**) Dendritic cell population. (**l**) Macrophage population.

C-ImmSim simulation results revealed a significant augmentation of primary immune responses following each vaccine administration. As illustrated in Fig. 12a, the levels of antibodies specific to the antigen (IgM, IgG1, and IgG2) significantly rose during the booster immune responses, which corresponded with a decline in antigen levels. The increased production of cytokines, such as IFN-γ and IL-2 (refer to Fig. 12b), was linked to heightened immune activation observed during the immunization process. These cytokines are pivotal in mediating cellular immune responses. The increased populations of activated B cells, helper T cells, as well as regulatory and cytotoxic T cells, indicate potentiated secondary immune reactions. Collectively, these results lead us to speculate that vaccination promotes efficient antigen clearance and generates immunological memory.

## Discussion

Syphilis, caused by *T. pallidum*, has increasing incidence rates reported worldwide^81^. Existing countermeasures, including screening and therapeutic approaches, are inadequate in addressing the rising infection rates^15^. For decades, penicillin has been the first-line antibiotic recommended for the treatment of syphilis and remains effective against *T. pallidum* infection^11^. However, while antibiotics can eliminate the pathogen, they do not prevent reinfection or reverse late-stage tissue damage. Furthermore, the persistently high prevalence of syphilis among high-risk populations underscores the limitations of traditional control measures, including sexual education and screening/treatment programs, in effectively disrupting transmission chains^82^. Consequently, it is essential to create a vaccine for syphilis that can halt the spread of this harmful bacterium. However, the creation of a syphilis vaccine faces numerous challenges, including difficulties in culturing *T. pallidum* in vitro, limitations in genetic manipulation techniques, and the pathogen’s complex immune evasion strategies^6^. As a result, no approved vaccine currently exists for the prevention of human syphilis, and research in this area remains at the experimental stage.

In the pursuit of syphilis vaccine development, earlier studies have explored HSPs and whole-genome-derived proteins as candidate antigens. However, HSPs are highly conserved across evolution and are ubiquitous in all organisms and cells, a characteristic that may contribute to the pathogenesis of autoimmune diseases^83–85^. Furthermore, vaccine strategies based on whole-genome-derived antigens face inherent limitations, as they encompass numerous components, including antigens with low immunogenicity or non-protective properties. For instance, in rabbits immunized with Tp0126, anti-Tp0126 antibodies were detectable only after a third booster immunization, underscoring the protein’s suboptimal immunogenicity and limited protective efficacy^86^. These challenges underscore the critical need to select optimal antigenic targets and to develop novel, highly specific immunogens that will advance syphilis vaccine research.

Adhesin proteins are promising vaccine targets due to their roles as key virulence factors and surface-exposed antigens. Research has demonstrated that inoculating rabbit models with the adhesins Tp0751 and Tp0136 can effectively prevent the spread of *T. pallidum*, underscoring the potential of these adhesins as candidate vaccines^19,87,88^. This study focuses on several syphilis adhesins: Tp0136, Tp0155, Tp0435, Tp0483, Tp0750, Tp0751, and Tp0954. These proteins exhibit diverse mechanisms that enable effective attachment to host tissues, dissemination, and colonization. They mediate bacterial adherence through interactions with host molecules, including fibronectin, laminin, fibrinogen/fibrin, and placental glycosaminoglycans^5^. Certain adhesins possess protease activity, degrading host barrier proteins to inhibit coagulation cascades and promote thrombolysis, thereby facilitating barrier penetration^89,90^. Notably, Tp0954 binds specific placental glycosaminoglycans, potentially driving placental colonization and maternal–fetal transmission^91–94^. However, single-adhesin vaccines encounter significant challenges: Tp0136 only delays disease progression in rabbit models, while data from natural infections reveal that 70% of patients fail to develop Tp0155-specific antibodies, indicating suboptimal immunogenicity^95–97^.

Given the limitations of single adhesin proteins in blocking *T. pallidum* adhesion, this vaccine development initiative explores the functional profiling of syphilis adhesins to identify highly immunogenic epitopes. We constructed a multi-epitope vaccine as a novel strategy that enables the host immune system to recognize epitopes from diverse adhesin proteins, thereby eliciting broader immune responses that enhance vaccine efficacy and protection. Recent advances in bioinformatics methodologies and the availability of accessible internet databases have created viable pathways for epitope selection and pre-evaluation, significantly expediting the vaccine development process. Compared to conventional approaches, reverse vaccinology offers a highly specific and cost-effective solution^98^. To facilitate the development of multi-epitope vaccines, seven adhesins from *T. pallidum* were selected as candidate proteins. Following an analysis of antigenicity, solubility, and physicochemical properties, B- and T-cell epitopes were screened. Epitopes were connected using KK, AAY, and GPGPG linkers, where AAY serves as a rigid spacer, while GPGPG and KK function as flexible spacers. Flexible linkers enhance protein solubility, whereas rigid linkers maintain structural stability.

The constructed multi-epitope vaccine underwent structural prediction, molecular dynamics simulation, and immunoinformatics simulation. Our predictions indicate that the resulting vaccine exhibits high antigenicity, solubility, and immunogenicity, along with non-allergenic and non-toxic properties and can be purified by Ni^2^⁺ affinity chromatography. Through 100 ns MD simulations, this study systematically evaluated the conformational stability and ligand-receptor energetics of the Vaccine-TLR2 and Vaccine-TLR4 complexes. Integrated trajectory analysis and quantitative metrics demonstrated favorable dynamic stability in both complexes; however, the Vaccine-TLR4 complex demonstrated moderately superior overall binding affinity. These findings suggest its enhanced potential to form stable and biologically effective complexes within biological systems^99^. The immunoinformatics simulation observed the immune system’s antibody response elicited by the introduction of the developed vaccine. This protein is capable of inducing high levels of IFN-γ secretion. Crucially, the Th1-type cellular response induced by adhesins plays a vital role in eliminating intracellularly colonized spirochetes. Following the initial injection, levels of interferon and IL-2 increased and remained elevated after subsequent antigen injections. This profile indicates a sufficient number of helper T cells, facilitating efficient antibody production and thus supporting a robust humoral immune response^100,101^. Furthermore, molecular docking experiments were conducted between the CTL and HTL epitopes and their corresponding HLA molecules. The results demonstrated strong binding affinity between these epitopes and the HLA molecules, suggesting their potential to activate cellular immune responses and their importance in the overall immune reaction. Leveraging the emerging potential of mRNA-based platforms for vaccination, gene therapy, and cancer immunotherapy, we developed an mRNA architecture that demonstrates enhanced stability, as indicated by an optimal secondary structure MFE of − 497.80 kcal/mol^102,103^. Therefore, these data suggest that the vaccine developed herein has the potential to elicit sustained cellular and humoral immune responses.

This vaccine targets multiple antigenic sites and is capable of generating robust and extensive immune responses. Unlike single-antigen vaccines, the multi-epitope vaccine effectively addresses the constraints posed by genetic diversity, thereby providing improved safety and adaptability. Although recent studies have identified novel antigenic proteins by scanning the complete genome of pathogens, our research focuses on adhesin proteins with well-validated functions^104,105^. The advantages of such targets lie in their clear mechanisms of action, which can effectively circumvent the unknown risks associated with novel antigenic proteins. However, this study also presents certain limitations. Firstly, the scope of epitope selection was restricted, necessitating prioritized choices. Additionally, the current study predominantly relies on in silico simulations, which may not fully reproduce the complex nature of immune responses observed in vivo*.* While the computational evaluation addressed the vaccine construct’s stability and antigenicity, additional experimental validation using immunological assays and testing in animal models will be essential to conclusively determine its safety and effectiveness.

## Conclusion

The escalating global incidence of syphilis has raised significant concerns among public health organizations, primarily due to the current lack of effective therapeutic interventions targeting the disease’s pathogenesis. Consequently, vaccination represents a more efficacious preventive strategy. This study developed a safe, highly antigenic, and non-allergenic multi-epitope adhesin protein. Diverging from conventional single-antigen approaches, our multiepitope design seeks to generate broader cross-protective immune responses. The multiplexed epitopes synergistically target immune receptors, including TLR2 and TLR4, thereby enabling broad-spectrum immune activation. Nevertheless, these findings and outcomes are currently supported solely by in silico simulations, highlighting the need for further animal studies to validate their practical efficacy.

## Supplementary Information

Below is the link to the electronic supplementary material.

Supplementary Material 1

## Data Availability

The datasets analysed during the current study are available in the GenBank repository, https://www.ncbi.nlm.nih.gov/nuccore/AE000520.1.

## References

[1] Du, M. et al. Increasing incidence rates of sexually transmitted infections from 2010 to 2019: An analysis of temporal trends by geographical regions and age groups from the 2019 global burden of disease study. *BMC. Infect. Dis.* **22**, 574. 10.1186/s12879-022-07544-7 (2022). 35754034 10.1186/s12879-022-07544-7 PMC9233762

[2] Lu, S. et al. Characterization of Treponema pallidum dissemination in C57BL/6 Mice. *Front Immunol.* **11**, 577129. 10.3389/fimmu.2020.577129 (2020). 33488577 10.3389/fimmu.2020.577129 PMC7819853

[3] Zhang, X. et al. Diagnostic importance of *Treponema pallidum* Tp0971 in the serological assessment of treatment efficacy for syphilis. *Ann. Clin. Microbiol. Antimicrob.* **24**, 58. 10.1186/s12941-025-00825-4 (2025). 41126282 10.1186/s12941-025-00825-4 PMC12542151

[4] Luo, X. et al. A preliminary study on the proinflammatory mechanisms of *Treponema pallidum* outer membrane protein Tp92 in human macrophages and HMEC-1 cells. *Microb. Pathog.* **110**, 176–183. 10.1016/j.micpath.2017.06.046 (2017). 28668606 10.1016/j.micpath.2017.06.046

[5] Djokic, V., Giacani, L. & Parveen, N. Analysis of host cell binding specificity mediated by the Tp0136 adhesin of the syphilis agent *Treponema pallidum* subsp. *pallidum*. *PLoS Negl. Trop. Dis.* **13**, e0007401. 10.1371/journal.pntd.0007401 (2019). 31071095 10.1371/journal.pntd.0007401 PMC6529012

[6] Tang, Y. et al. Investigation of the immune escape mechanism of *Treponema pallidum*. *Infection* **51**, 305–321. 10.1007/s15010-022-01939-z (2023). 36260281 10.1007/s15010-022-01939-z

[7] Zuo, W., Xiao, Y., Xiang, Q., Xiao, S. & Xie, Y. Participants in *Treponema pallidum* pathogenesis: Progress in functional proteins. *Front. Immunol.* **16**, 1632677. 10.3389/fimmu.2025.1632677 (2025). 40933996 10.3389/fimmu.2025.1632677 PMC12417408

[8] Ávila-Nieto, C. et al. Syphilis vaccine: Challenges, controversies and opportunities. *Front. Immunol.* **14**, 1126170. 10.3389/fimmu.2023.1126170 (2023). 37090699 10.3389/fimmu.2023.1126170 PMC10118025

[9] Liu, Z. et al. *Treponema pallidum* inhibits CD4+ T-cell proliferation through METAP2: Insights from Mendelian randomization analysis. *AMB Express* **15**, 126. 10.1186/s13568-025-01940-3 (2025). 40853519 10.1186/s13568-025-01940-3 PMC12379665

[10] Lu, S. et al. *Treponema pallidum* Tp0751 alters the expression of tight junction proteins by promoting bEnd3 cell apoptosis and IL-6 secretion. *Int. J. Med. Microbiol.* **312**, 151553. 10.1016/j.ijmm.2022.151553 (2022). 35358795 10.1016/j.ijmm.2022.151553

[11] Jaiswal, A. K., Rodrigues Gomes, L. G., Ferreira Maciel de Oliveira, A., de Castro Soares, S. & Azevedo, V. The critical role of penicillin in syphilis treatment and emerging resistance challenges. *Diseases* **13**, 020041. 10.3390/diseases13020041 (2025). 10.3390/diseases13020041 PMC11854480 39997048

[12] Chevalier, F. J., Bacon, O., Johnson, K. A. & Cohen, S. E. Syphilis: A review. *JAMA* **334**, 1927–1940. 10.1001/jama.2025.17362 (2025). 41100079 10.1001/jama.2025.17362

[13] Tantalo, L. C. et al. Antimicrobial susceptibility of *Treponema pallidum* subspecies *pallidum*: An in-vitro study. *Lancet Microbe* **4**, e994–e1004. 10.1016/s2666-5247(23)00219-7 (2023). 37827185 10.1016/S2666-5247(23)00219-7 PMC10686905

[14] Chen, J., Huang, J., Liu, Z. & Xie, Y. *Treponema pallidum* outer membrane proteins: Current status and prospects. *Pathog. Dis.* **80**, ftac023. 10.1093/femspd/ftac023 (2022). 35869970 10.1093/femspd/ftac023

[15] Waugh, S. & Cameron, C. E. Syphilis vaccine development: Aligning vaccine design with manufacturing requirements. *Hum. Vaccin. Immunother.* **20**, 2399915. 10.1080/21645515.2024.2399915 (2024). 39262177 10.1080/21645515.2024.2399915 PMC11404580

[16] Jiang, C. et al. Evaluation of FlaB1, FlaB2, FlaB3, and Tp0463 of *Treponema pallidum* for serodiagnosis of syphilis. *Diagn. Microbiol. Infect. Dis.* **84**, 105–111. 10.1016/j.diagmicrobio.2015.10.005 (2016). 26607421 10.1016/j.diagmicrobio.2015.10.005

[17] Jiang, J. et al. A comprehensive strategy for the development of a multi-epitope vaccine targeting *Treponema pallidum*, utilizing heat shock proteins, encompassing the entire process from vaccine design to in vitro evaluation of immunogenicity. *Front. Microbiol.* **16**, 1551437. 10.3389/fmicb.2025.1551437 (2025). 40177491 10.3389/fmicb.2025.1551437 PMC11962626

[18] Li, S., Li, W., Jin, Y., Wu, B. & Wu, Y. Advancements in the development of nucleic acid vaccines for syphilis prevention and control. *Hum. Vaccin. Immunother.* **19**, 2234790. 10.1080/21645515.2023.2234790 (2023). 37538024 10.1080/21645515.2023.2234790 PMC10405752

[19] Lithgow, K. V. et al. A defined syphilis vaccine candidate inhibits dissemination of *Treponema pallidum* subspecies *pallidum*. *Nat. Commun.* **8**, 14273. 10.1038/ncomms14273 (2017). 28145405 10.1038/ncomms14273 PMC5296639

[20] Zheng, K. et al. Immunogenicity and protective efficacy against *Treponema pallidum* in New Zealand rabbits immunized with plasmid DNA encoding flagellin. *Emerg. Microbes Infect.* **7**, 177. 10.1038/s41426-018-0176-0 (2018). 30405111 10.1038/s41426-018-0176-0 PMC6220273

[21] Jun, T. et al. Immunisation with the glycolytic enzyme enolase inhibits dissemination of *Treponema pallidum* in C57BL/6 mice. *Microb. Pathog.* **184**, 106374. 10.1016/j.micpath.2023.106374 (2023). 37802159 10.1016/j.micpath.2023.106374

[22] Goodswen, S. J., Kennedy, P. J. & Ellis, J. T. A guide to current methodology and usage of reverse vaccinology towards in silico vaccine discovery. *FEMS Microbiol. Rev.*10.1093/femsre/fuad004 (2023). 36806618 10.1093/femsre/fuad004

[23] Fan, X. et al. Construction and immunogenicity of a T cell epitope-based subunit vaccine candidate against *Mycobacterium tuberculosis*. *Vaccine* **39**, 6860–6865. 10.1016/j.vaccine.2021.10.034 (2021). 34702619 10.1016/j.vaccine.2021.10.034

[24] Wang, C. et al. Evaluation of a tandem *Chlamydia psittaci* Pgp3 multiepitope peptide vaccine against a pulmonary chlamydial challenge in mice. *Microb. Pathog.* **147**, 104256. 10.1016/j.micpath.2020.104256 (2020). 32416138 10.1016/j.micpath.2020.104256

[25] Yu, J. et al. A novel multi-component protein vaccine ECP001 containing a protein polypeptide antigen nPstS1 riching in T-cell epitopes showed good immunogenicity and protection in mice. *Front. Immunol.* **14**, 1138818. 10.3389/fimmu.2023.1138818 (2023). 37153610 10.3389/fimmu.2023.1138818 PMC10161251

[26] Xu, L. et al. Development of a novel multi-epitope vaccine against *Ureaplasma urealyticum* infection through reverse vaccinology approach. *Mol. Divers.*10.1007/s11030-025-11234-2 (2025). 40537712 10.1007/s11030-025-11234-2

[27] Xu, L. et al. Development of a novel multi-epitope vaccine against *Streptococcus anginosus* infection via reverse vaccinology approach. *Immunology* **175**, 339–358. 10.1111/imm.13936 (2025). 40267989 10.1111/imm.13936

[28] Isaacs, D. & McVernon, J. Introducing a new group B meningococcus vaccine. *BMJ* **348**, g2415. 10.1136/bmj.g2415 (2014). 24696308 10.1136/bmj.g2415

[29] Duffy, J. et al. Safety surveillance of bivalent meningococcal group B vaccine, vaccine adverse event reporting system, 2014-2018. *Open Forum Infect. Dis.* **7**, ofaa516. 10.1093/ofid/ofaa516 (2020). 33324721 10.1093/ofid/ofaa516 PMC7724509

[30] Basmenj, E. R. et al. Computational epitope-based vaccine design with bioinformatics approach; A review. *Heliyon* **11**, e41714. 10.1016/j.heliyon.2025.e41714 (2025). 39866399 10.1016/j.heliyon.2025.e41714 PMC11761309

[31] Altschul, S. F., Gish, W., Miller, W., Myers, E. W. & Lipman, D. J. Basic local alignment search tool. *J. Mol. Biol.* **215**, 403–410. 10.1016/s0022-2836(05)80360-2 (1990). 2231712 10.1016/S0022-2836(05)80360-2

[32] Clem, A. S. Fundamentals of vaccine immunology. *J. Glob. Infect. Dis.* **3**, 73–78. 10.4103/0974-777x.77299 (2011). 21572612 10.4103/0974-777X.77299 PMC3068582

[33] Saha, S. & Raghava, G. P. Prediction of continuous B-cell epitopes in an antigen using recurrent neural network. *Proteins* **65**, 40–48. 10.1002/prot.21078 (2006). 16894596 10.1002/prot.21078

[34] Doytchinova, I. A. & Flower, D. R. VaxiJen: A server for prediction of protective antigens, tumour antigens and subunit vaccines. *BMC Bioinformatics* **8**, 4. 10.1186/1471-2105-8-4 (2007). 17207271 10.1186/1471-2105-8-4 PMC1780059

[35] Gupta, S. et al. In silico approach for predicting toxicity of peptides and proteins. *PLoS ONE* **8**, e73957. 10.1371/journal.pone.0073957 (2013). 24058508 10.1371/journal.pone.0073957 PMC3772798

[36] Dimitrov, I., Bangov, I., Flower, D. R. & Doytchinova, I. AllerTOP v.2--A server for in silico prediction of allergens. *J. Mol. Model.* **20**, 2278. 10.1007/s00894-014-2278-5 (2014). 24878803 10.1007/s00894-014-2278-5

[37] Yan, Z. et al. Next-generation IEDB tools: A platform for epitope prediction and analysis. *Nucleic Acids Res.* **52**, W526-w532. 10.1093/nar/gkae407 (2024). 38783079 10.1093/nar/gkae407 PMC11223806

[38] Calis, J. J. et al. Properties of MHC class I presented peptides that enhance immunogenicity. *PLoS Comput. Biol.* **9**, e1003266. 10.1371/journal.pcbi.1003266 (2013). 24204222 10.1371/journal.pcbi.1003266 PMC3808449

[39] Wang, P. et al. Peptide binding predictions for HLA DR, DP and DQ molecules. *BMC Bioinformatics* **11**, 568. 10.1186/1471-2105-11-568 (2010). 21092157 10.1186/1471-2105-11-568 PMC2998531

[40] Dhanda, S. K., Vir, P. & Raghava, G. P. Designing of interferon-gamma inducing MHC class-II binders. *Biol. Direct.* **8**, 30. 10.1186/1745-6150-8-30 (2013). 24304645 10.1186/1745-6150-8-30 PMC4235049

[41] Roohparvar Basmenj, E., Omidvar, B., Kiumarsy, A., Izadkhah, H. & Ghiabi, S. Design of a multi-epitope-based peptide vaccine against the SARS-CoV-2 Omicron variant using bioinformatics approach. *J. Biomol. Struct. Dyn.* **42**, 7945–7956. 10.1080/07391102.2023.2241926 (2024). 37539837 10.1080/07391102.2023.2241926

[42] Bui, H. H. et al. Predicting population coverage of T-cell epitope-based diagnostics and vaccines. *BMC Bioinformatics* **7**, 153. 10.1186/1471-2105-7-153 (2006). 16545123 10.1186/1471-2105-7-153 PMC1513259

[43] Maupetit, J., Derreumaux, P. & Tufféry, P. A fast method for large-scale de novo peptide and miniprotein structure prediction. *J. Comput. Chem.* **31**, 726–738. 10.1002/jcc.21365 (2010). 19569182 10.1002/jcc.21365

[44] Berman, H. M. The protein data bank. *Nucleic Acids Res.* **28**, 235–242. 10.1093/nar/28.1.235 (2000). 10592235 10.1093/nar/28.1.235 PMC102472

[45] Weng, G. et al. HawkDock: A web server to predict and analyze the protein-protein complex based on computational docking and MM/GBSA. *Nucleic Acids Res.* **47**, W322-w330. 10.1093/nar/gkz397 (2019). 31106357 10.1093/nar/gkz397 PMC6602443

[46] Tan, C. et al. Development of multi-epitope mRNA vaccine against *Clostridioides difficile* using reverse vaccinology and immunoinformatics approaches. *Synth. Syst. Biotechnol.* **9**, 667–683. 10.1016/j.synbio.2024.05.008 (2024). 38817826 10.1016/j.synbio.2024.05.008 PMC11137598

[47] Guo, H. et al. Effect of flexible linker length on the activity of fusion protein 4-coumaroyl-CoA ligase::stilbene synthase. *Mol. Biosyst.* **13**, 598–606. 10.1039/c6mb00563b (2017). 28181620 10.1039/c6mb00563b

[48] Zhao, T. et al. Vaccine adjuvants: Mechanisms and platforms. *Signal Transduct. Target. Ther.* **8**, 283. 10.1038/s41392-023-01557-7 (2023). 37468460 10.1038/s41392-023-01557-7 PMC10356842

[49] Arai, R., Ueda, H., Kitayama, A., Kamiya, N. & Nagamune, T. Design of the linkers which effectively separate domains of a bifunctional fusion protein. *Protein Eng.* **14**, 529–532. 10.1093/protein/14.8.529 (2001). 11579220 10.1093/protein/14.8.529

[50] Liu, J. et al. A multi-epitope subunit vaccine providing broad cross-protection against diverse serotypes of *Streptococcus suis*. *npj Vaccines* **9**, 216. 10.1038/s41541-024-01015-7 (2024). 39543108 10.1038/s41541-024-01015-7 PMC11564553

[51] Hebditch, M., Carballo-Amador, M. A., Charonis, S., Curtis, R. & Warwicker, J. Protein-Sol: A web tool for predicting protein solubility from sequence. *Bioinformatics* **33**, 3098–3100. 10.1093/bioinformatics/btx345 (2017). 28575391 10.1093/bioinformatics/btx345 PMC5870856

[52] Conchillo-Solé, O. et al. AGGRESCAN: A server for the prediction and evaluation of “hot spots” of aggregation in polypeptides. *BMC Bioinformatics* **8**, 65. 10.1186/1471-2105-8-65 (2007). 17324296 10.1186/1471-2105-8-65 PMC1828741

[53] Mészáros, B., Erdos, G. & Dosztányi, Z. IUPred2A: Context-dependent prediction of protein disorder as a function of redox state and protein binding. *Nucleic Acids Res.* **46**, W329-w337. 10.1093/nar/gky384 (2018). 29860432 10.1093/nar/gky384 PMC6030935

[54] Wilkins, M. R. et al. Protein identification and analysis tools in the ExPASy server. *Methods Mol Biol.* **112**, 531–552. 10.1385/1-59259-584-7:531 (1999). 10027275 10.1385/1-59259-584-7:531

[55] Nielsen, H., Teufel, F., Brunak, S. & von Heijne, G. SignalP: The evolution of a web server. *Methods Mol. Biol.* **2836**, 331–367. 10.1007/978-1-0716-4007-4_17 (2024). 38995548 10.1007/978-1-0716-4007-4_17

[56] Jones, D. T. Protein secondary structure prediction based on position-specific scoring matrices. *J. Mol. Biol.* **292**, 195–202. 10.1006/jmbi.1999.3091 (1999). 10493868 10.1006/jmbi.1999.3091

[57] Geourjon, C. & Deléage, G. SOPMA: Significant improvements in protein secondary structure prediction by consensus prediction from multiple alignments. *Comput. Appl. Biosci.* **11**, 681–684. 10.1093/bioinformatics/11.6.681 (1995). 8808585 10.1093/bioinformatics/11.6.681

[58] Adam, K. M. Immunoinformatics approach for multi-epitope vaccine design against structural proteins and ORF1a polyprotein of severe acute respiratory syndrome coronavirus-2 (SARS-CoV-2). *Trop Dis Travel Med Vaccines* **7**, 22. 10.1186/s40794-021-00147-1 (2021). 34238372 10.1186/s40794-021-00147-1 PMC8266167

[59] Abramson, J. et al. Accurate structure prediction of biomolecular interactions with AlphaFold 3. *Nature* **630**, 493–500. 10.1038/s41586-024-07487-w (2024). 38718835 10.1038/s41586-024-07487-w PMC11168924

[60] Pourseif, M. M., Baradaran Hosseini, S. A., Khoshraftar, S. H. & Omidi, Y. The role of bioinformatics algorithms in modern biopharmaceutical design: Progress, challenges, and future perspectives. *Bioimpacts* **15**, 33072. 10.34172/bi.33072 (2025). 41409583 10.34172/bi.33072 PMC12705280

[61] Heo, L., Park, H. & Seok, C. GalaxyRefine: Protein structure refinement driven by side-chain repacking. *Nucleic Acids Res.* **41**, W384-388. 10.1093/nar/gkt458 (2013). 23737448 10.1093/nar/gkt458 PMC3692086

[62] Wiederstein, M. & Sippl, M. J. ProSA-web: Interactive web service for the recognition of errors in three-dimensional structures of proteins. *Nucleic Acids Res.* **35**, W407-410. 10.1093/nar/gkm290 (2007). 17517781 10.1093/nar/gkm290 PMC1933241

[63] Sippl, M. J. Recognition of errors in three-dimensional structures of proteins. *Proteins* **17**, 355–362. 10.1002/prot.340170404 (1993). 8108378 10.1002/prot.340170404

[64] Colovos, C. & Yeates, T. O. Verification of protein structures: Patterns of nonbonded atomic interactions. *Protein Sci.* **2**, 1511–1519. 10.1002/pro.5560020916 (1993). 8401235 10.1002/pro.5560020916 PMC2142462

[65] Kang, S. M. & Compans, R. W. Host responses from innate to adaptive immunity after vaccination: Molecular and cellular events. *Mol. Cells* **27**, 5–14. 10.1007/s10059-009-0015-1 (2009). 19214429 10.1007/s10059-009-0015-1 PMC6280669

[66] Huang, T. et al. MicroRNA-101-3p downregulates TLR2 expression, leading to reduction in cytokine production by *Treponema pallidum*-stimulated macrophages. *J. Invest. Dermatol.* **140**, 1566-1575.e1561. 10.1016/j.jid.2019.12.012 (2020). 31930972 10.1016/j.jid.2019.12.012

[67] Peng, R. R., Shang, S. X., Zhao, L. S. & Long, F. Q. MiR-216a-5p-containing exosomes suppress rTp17-induced inflammatory response by targeting TLR4. Biosci. Rep. 10.1042/bsr20190686 (2019). 10.1042/BSR20190686 PMC6684949 31358689

[68] Laskowski, R. A., Jabłońska, J., Pravda, L., Vařeková, R. S. & Thornton, J. M. PDBsum: Structural summaries of PDB entries. *Protein Sci.* **27**, 129–134. 10.1002/pro.3289 (2018). 28875543 10.1002/pro.3289 PMC5734310

[69] Abraham, M. J. et al. GROMACS: High performance molecular simulations through multi-level parallelism from laptops to supercomputers. *SoftwareX* **1–2**, 19–25. 10.1016/j.softx.2015.06.001 (2015).

[70] Van Der Spoel, D. et al. GROMACS: Fast, flexible, and free. *J. Comput. Chem.* **26**, 1701–1718. 10.1002/jcc.20291 (2005). 16211538 10.1002/jcc.20291

[71] López-Blanco, J. R., Aliaga, J. I., Quintana-Ortí, E. S. & Chacón, P. iMODS: Internal coordinates normal mode analysis server. *Nucleic Acids Res.* **42**, W271-276. 10.1093/nar/gku339 (2014). 24771341 10.1093/nar/gku339 PMC4086069

[72] Ponomarenko, J. et al. ElliPro: A new structure-based tool for the prediction of antibody epitopes. *BMC Bioinformatics* **9**, 514. 10.1186/1471-2105-9-514 (2008). 19055730 10.1186/1471-2105-9-514 PMC2607291

[73] Puigbò, P., Guzmán, E., Romeu, A. & Garcia-Vallvé, S. OPTIMIZER: A web server for optimizing the codon usage of DNA sequences. *Nucleic Acids Res.* **35**, W126-131. 10.1093/nar/gkm219 (2007). 17439967 10.1093/nar/gkm219 PMC1933141

[74] Quax, T. E., Claassens, N. J., Söll, D. & van der Oost, J. Codon Bias as a means to fine-tune gene expression. *Mol Cell.* **59**, 149–161. 10.1016/j.molcel.2015.05.035 (2015). 26186290 10.1016/j.molcel.2015.05.035 PMC4794256

[75] Suneesh, N. S. et al. Reverse vaccinology-based design of multivalent multiepitope mRNA vaccines targeting key viral proteins of herpes simplex virus type-2. *Front Immunol.* **16**, 1586271. 10.3389/fimmu.2025.1586271 (2025). 40463368 10.3389/fimmu.2025.1586271 PMC12130045

[76] Reshetnikov, V. et al. Untranslated region sequences and the efficacy of mRNA vaccines against tuberculosis. *Int. J. Mol. Sci.*10.3390/ijms25020888 (2024). 38255961 10.3390/ijms25020888 PMC10815675

[77] Nance, K. D. & Meier, J. L. Modifications in an emergency: The role of N1-Methylpseudouridine in COVID-19 vaccines. *ACS Cent. Sci.* **7**, 748–756. 10.1021/acscentsci.1c00197 (2021). 34075344 10.1021/acscentsci.1c00197 PMC8043204

[78] Mathews, D. H. et al. Incorporating chemical modification constraints into a dynamic programming algorithm for prediction of RNA secondary structure. *Proc Natl Acad Sci U S A.* **101**, 7287–7292. 10.1073/pnas.0401799101 (2004). 15123812 10.1073/pnas.0401799101 PMC409911

[79] Rapin, N., Lund, O., Bernaschi, M. & Castiglione, F. Computational immunology meets bioinformatics: the use of prediction tools for molecular binding in the simulation of the immune system. *PLoS ONE* **5**, e9862. 10.1371/journal.pone.0009862 (2010). 20419125 10.1371/journal.pone.0009862 PMC2855701

[80] Rapin, N., Lund, O. & Castiglione, F. Immune system simulation online. *Bioinformatics* **27**, 2013–2014. 10.1093/bioinformatics/btr335 (2011). 21685045 10.1093/bioinformatics/btr335

[81] Xiao, Y. et al. Molecular subtyping and surveillance of resistance genes in *Treponema pallidum* DNA from patients with secondary and latent syphilis in Hunan, China. *Sex. Transm. Dis.* **43**, 310–316. 10.1097/olq.0000000000000445 (2016). 27100768 10.1097/OLQ.0000000000000445

[82] Luo, Y., Xie, Y. & Xiao, Y. Laboratory diagnostic tools for syphilis: Current status and future prospects. *Front. Cell Infect. Microbiol.* **10**, 574806. 10.3389/fcimb.2020.574806 (2020). 33628742 10.3389/fcimb.2020.574806 PMC7897658

[83] Hagymasi, A. T., Dempsey, J. P. & Srivastava, P. K. Heat-shock proteins. *Curr. Protoc.* **2**, e592. 10.1002/cpz1.592 (2022). 36367390 10.1002/cpz1.592

[84] Dreiza, C. M. et al. The small heat shock protein, HSPB6, in muscle function and disease. *Cell Stress Chaperones* **15**, 1–11. 10.1007/s12192-009-0127-8 (2010). 19568960 10.1007/s12192-009-0127-8 PMC2866971

[85] Zininga, T., Ramatsui, L. & Shonhai, A. Heat shock proteins as immunomodulants. *Molecules*10.3390/molecules23112846 (2018). 30388847 10.3390/molecules23112846 PMC6278532

[86] Haynes, A. M., Godornes, C., Ke, W. & Giacani, L. Evaluation of the protective ability of the *Treponema pallidum* subsp. *pallidum* Tp0126 OmpW homolog in the rabbit model of syphilis. *Infect. Immun.*10.1128/iai.00323-19 (2019). 31182617 10.1128/IAI.00323-19 PMC6652746

[87] Xu, M. et al. Two potential syphilis vaccine candidates inhibit dissemination of *Treponema pallidum*. *Front. Immunol.* **12**, 759474. 10.3389/fimmu.2021.759474 (2021). 34899710 10.3389/fimmu.2021.759474 PMC8657604

[88] He, Y. et al. Immunization with Tp0954, an adhesin of *Treponema pallidum*, provides protective efficacy in the rabbit model of experimental syphilis. *Front. Immunol.* **14**, 1130593. 10.3389/fimmu.2023.1130593 (2023). 36993963 10.3389/fimmu.2023.1130593 PMC10042077

[89] Houston, S. et al. The multifunctional role of the pallilysin-associated *Treponema pallidum* protein, Tp0750, in promoting fibrinolysis and extracellular matrix component degradation. *Mol. Microbiol.* **91**, 618–634. 10.1111/mmi.12482 (2014). 24303899 10.1111/mmi.12482 PMC3954913

[90] Houston, S. et al. Bifunctional role of the *Treponema pallidum* extracellular matrix binding adhesin Tp0751. *Infect. Immun.* **79**, 1386–1398. 10.1128/iai.01083-10 (2011). 21149586 10.1128/IAI.01083-10 PMC3067502

[91] Primus, S., Rocha, S. C., Giacani, L. & Parveen, N. Identification and functional assessment of the first placental adhesin of *Treponema pallidum* that may play critical role in congenital syphilis. *Front. Microbiol.* **11**, 621654. 10.3389/fmicb.2020.621654 (2020). 33408711 10.3389/fmicb.2020.621654 PMC7779807

[92] Giri, T. K. & Tollefsen, D. M. Placental dermatan sulfate: Isolation, anticoagulant activity, and association with heparin cofactor II. *Blood* **107**, 2753–2758. 10.1182/blood-2005-09-3755 (2006). 16339402 10.1182/blood-2005-09-3755 PMC1895383

[93] Suga, N. et al. Heparin/heparan sulfate/CD44-v3 enhances cell migration in term placenta-derived immortalized human trophoblast cells. *Biol. Reprod.* **86**, 131–138. 10.1095/biolreprod.111.093690 (2012). 22321833 10.1095/biolreprod.111.093690

[94] Li, W. et al. Research progress on the mechanism of *Treponema pallidum* breaking through placental barrier. *Microb. Pathog.* **185**, 106392. 10.1016/j.micpath.2023.106392 (2023). 37852552 10.1016/j.micpath.2023.106392

[95] Li, Q. L. et al. Screening the B- and T-cell epitope map of TP0136 and exploring their effect in a *Treponema pallidum* rabbit model. *Biomed. Pharmacother.* **167**, 115628. 10.1016/j.biopha.2023.115628 (2023). 37804809 10.1016/j.biopha.2023.115628

[96] Tomson, F. L., Conley, P. G., Norgard, M. V. & Hagman, K. E. Assessment of cell-surface exposure and vaccinogenic potentials of *Treponema pallidum* candidate outer membrane proteins. *Microbes Infect.* **9**, 1267–1275. 10.1016/j.micinf.2007.05.018 (2007). 17890130 10.1016/j.micinf.2007.05.018 PMC2112743

[97] Van Voorhis, W. C. et al. Serodiagnosis of syphilis: Antibodies to recombinant Tp0453, Tp92, and Gpd proteins are sensitive and specific indicators of infection by *Treponema pallidum*. *J. Clin. Microbiol.* **41**, 3668–3674. 10.1128/jcm.41.8.3668-3674.2003 (2003). 12904373 10.1128/JCM.41.8.3668-3674.2003 PMC179844

[98] Rappuoli, R., Bottomley, M. J., D’Oro, U., Finco, O. & De Gregorio, E. Reverse vaccinology 2.0: Human immunology instructs vaccine antigen design. *J. Exp. Med.* **213**, 469–481. 10.1084/jem.20151960 (2016). 27022144 10.1084/jem.20151960 PMC4821650

[99] Xie, Y. et al. *Treponema pallidum* flagellin FlaA2 induces IL-6 secretion in THP-1 cells via the Toll-like receptor 2 signaling pathway. *Mol. Immunol.* **81**, 42–51. 10.1016/j.molimm.2016.11.005 (2017). 27888719 10.1016/j.molimm.2016.11.005

[100] Shantier, S. W. et al. Novel multi epitope-based vaccine against monkeypox virus: Vaccinomic approach. *Sci. Rep.* **12**, 15983. 10.1038/s41598-022-20397-z (2022). 36156077 10.1038/s41598-022-20397-z PMC9510130

[101] Aiman, S. et al. Multi-epitope chimeric vaccine design against emerging Monkeypox virus via reverse vaccinology techniques- A bioinformatics and immunoinformatics approach. *Front. Immunol.* **13**, 985450. 10.3389/fimmu.2022.985450 (2022). 36091024 10.3389/fimmu.2022.985450 PMC9452969

[102] Tan, T. et al. mRNA vaccine - A new cancer treatment strategy. *Curr. Cancer Drug Targets* **23**, 669–681. 10.2174/1568009623666230222124424 (2023). 36809966 10.2174/1568009623666230222124424

[103] Jin, Y., Hou, C., Li, Y., Zheng, K. & Wang, C. mRNA vaccine: How to meet the challenge of SARS-CoV-2. *Front. Immunol.* **12**, 821538. 10.3389/fimmu.2021.821538 (2021). 35126377 10.3389/fimmu.2021.821538 PMC8813741

[104] Roohparvar Basmenj, E. et al. A novel approach to design a multiepitope peptide as a vaccine candidate for *Bordetella pertussis*. *J. Biomol. Struct. Dyn.* **42**, 13738–13750. 10.1080/07391102.2023.2278081 (2024). 37937610 10.1080/07391102.2023.2278081

[105] Koupaei, F. N. et al. Design of a multi-epitope vaccine candidate against *Vibrio cholerae*. *Sci. Rep.* **15**, 11033. 10.1038/s41598-025-90598-9 (2025). 40164630 10.1038/s41598-025-90598-9 PMC11958690

