# Supplementary material for: Designing a novel multiepitope vaccine candidate against Treponema pallidum via adhesins using reverse vaccinology
Source: Sci Rep. 2026 Apr 1;16:15305. doi: 10.1038/s41598-026-45084-1 (PMC13181057; doi:10.1038/s41598-026-45084-1)
Supplement: Supplementary file 1 — Supplementary Material 1 [file 41598_2026_45084_MOESM1_ESM.pdf]

Supplementary Material

Designing a novel multiepitope vaccine candidate against *Treponema pallidum* via adhesins using reverse vaccinology

Hongmei Tang[1，5†], Zhixi Chen[1，3†], Hongxia Yan[4], Zhen He[5], Ranhui Li[5], Yafeng Xie[2*], Xiaoliu Wang[1*]

[1]Department of Dermatology and Venereology, The First Affiliated Hospital, Hengyang Medical College, University of South China, Hengyang 421001, Hunan, China.

[2]Department of Clinical Laboratory, The Second Affiliated Hospital, Hengyang Medical School, University of South China, Hengyang, 421001, Hunan, China.

[3]Department of Blood Transfusion, the First Affiliated Hospital, Hengyang Medical College, University of South China, Hengyang, 421001, Hunan, China.

[4]Department of Pediatrics, The Second Affiliated Hospital, Hengyang Medical School, University of South China, Hengyang, 421001 Hunan, China.

[5]Institute of Pathogenic Biology,Basic Medical School, Hengyang Medical College, Key Laboratory of Special Pathogen Prevention and Control of Hunan Province, University of South China, Hengyang 421001, China

*** Correspondence:**

Yafeng Xie: 2016020062@usc.edu.cn

Xiaoliu Wang: wangxiaoliu@usc.edu.cn

The first author Email:

Hongmei Tang:20242023111613@stu.usc.edu.cn

Zhixi Chen: chenzhixi1982@sina.cn

Other author Email:

Hongxia Yan:2002020003@usc.edu.cn

Ranhui Li: liranhui@usc.edu.cn

Zhen He: 2803067796@qq.com

Table S1 Websites used in this research.

Parameter	Server Name	Website Address
Protein retrieval	NCBI	https://www.ncbi.nlm.nih.gov(accessed on 5 June 2025)
Antigenicity	Vaxijen 2.0	https://www.ddg-pharmfac.net/vaxijen(accessed on 10 June 2025)
Solubility	Protein-Sol	https://www.protein-sol.manchester.ac.uk(accessed on 10 June 2025)
B-cell prediction	ABCpred	http://www.webs.iiitd.edu.in/raghava/abcpred/(accessed on 12 June 2025)
Allergenicity	AllerTOP v2.1	https://www.ddg-pharmfac.net/AllerTOP/(accessed on 12 June 2025)
Toxicity	ToxinPred	http://crdd.osdd.net/raghava/toxinpred(accessed on 12 June 2025)
CTL prediction	IEDB	http://tools.iedb.org/mhci/(accessed on 20 June 2025)
HTL prediction	IEDB	http://tools.iedb.org/mhcii/(accessed on 27 June 2025)
Immunogenicity	IEDB	http://tools.iedb.org/immunogenicity/(accessed on 20 June 2025)
IFN-γ prediction	IFNepitope	http://crdd.osdd.net/raghava/ifnepitope/(accessed on 27 June 2025)
Aggregation prediction	aggrescan	http://bioinf.uab.es/aggrescan/(accessed on 8 July 2025)
Disorder prediction	IUPred	https://iupred2a.elte.hu/plot_new(accessed on 8 July 2025)
Physicochemical	Expasy	https://web.expasy.org/protparam/(accessed on 8 July 2025)
Transmembrane topology	DeepTMHMM	https://services.healthtech.dtu.dk/services/(accessed on 8 July 2025)
Signal peptide	SignalIP 6.0	https://services.healthtech.dtu.dk/services/(accessed on 8 July 2025)
Secondary structure	PSIPREDV3.3	http://bioinf.cs.ucl.ac.uk/psipred/(accessed on 10 July 2025)
Secondary structure	SOPMA	https://npsa.lyon.inserm.fr/cgi-bin/npsa_automat.pl?page=/NPSA/npsa_sopma.html(accessed on 10 July 2025)
3D structure	AlphaFold 3	https://alphafoldserver.com(accessed on 12 June 2025)
Structure refinement	GalaxyRefine	https://galaxy.seoklab.org/cgi-bin/submit.cgi?type=REFINE(accessed on 15 July 2025)
Z-score	ProSA-web	https://prosa.services.came.sbg.ac.at/prosa.php(accessed on 17 July 2025)
Ramachandran plot	SAVESv6.1	https://saves.mbi.ucla.edu(accessed on 17 July 2025)
Protein docking	HawkDock	http://cadd.zju.edu.cn/hawkdock/(accessed on 20 July 2025)
Protein–protein	PDBsum	https://www.ebi.ac.uk/thornton-srv/databases/pdbsum/(accessed on 23

interaction		July 2025)
Molecular dynamic simulation	iMODS	https//imods.iqfr.csic.es/(accessed on 25 June 2025)
Molecular dynamic simulation	Gromacs2022.3	https://manual.gromacs.org/(accessed on 11 July 2025)
Codon optimization	Optimizer	https://genomes.urv.es/OPTIMIZER/(accessed on 1 August 2025)
Cloning	SnapGene	https://www.snapgene.com/(accessed on 11 August 2025)
Immune simulation	C-ImmSim	https://kraken.iac.rm.cnr.it/C-IMMSIM/(accessed on 15 August 2025)
RNA prediction	RNAfold	https://rna.tbi.univie.ac.at//cgi-bin/RNAWebSuite/RNAfold.cgi(accessed on 17 August 2025)
Peptide structure	PEP-FOLD3	https://bioserv.rpbs.univ-paris-diderot.fr/services/PEP-FOLD3/(accessed on 11 August 2025)
Protein structure	protein Data Bank	https://www.rcsb.org(accessed on 11 August 2025)
Structure visualization	PyMOL	https://pymol.org/(accessed on 19 August 2025)
Conformational epitope prediction	ElliPro	http://tools.iedb.org/ellipro(accessed on 20 December 2025)
Conserved epitope	Epitopt Tool	https://bioinfcamptools.ir/Conserved-epitope-finder/upload.html(accessed on 22 December 2025)
Population coverage	IEDB	https://tools.iedb.org/population/(accessed on 20 December 2025)

Table S2 Conserved epitope analysis.

Protein	Epitope	Conserved percentage
Tp0136	TSSTQRPDLYAAVGES	56%
	EQYRGTVGR	98%
	QKIYVVEKNGGGNGV	84%
Tp0155	TGRSTGPHLHFTIYKN	13%
	GPHLHFTIY	13%
	ALLLFVTLL	13%
Tp0435	VSSEQSKAPHEKELYE	83%
	YMGAPGAGK	75%
Tp0483	LKTGSYTLRAITPRNI	81%
	YLYELYPRI	81%
	KADEAGAYV	83%
	KEQEARISW	78%
	VKPSFTGVSLQQTPS	56%
Tp0750	DLEHDAPLTSKYRGKQ	70%
	VPVDIFLMI	60%
	ALCTFLIHL	50%
Tp0751	SGSSTTTDPRSHGNAP	73%
	VAAWALYIF	60%
	IHVRAVEDVARLKIG	87%
Tp0954	TGSNSARESERAQLLK	33%
	YSFYLAFFY	42%
	LQLFDTLSPEHRAEK	38%

Table S3 Epitope sequence arrangement results.

Vaccine	Antigenicity	Instability index	Solubility	Aggregation	Disorder	Sequence identity (%)
MEVTP-1	1.0296	34.75	0.532	-4.30	0.3336475	0%
MEVTP-2	1.0025	34.53	0.532	-4.30	0.30425825	54.4%
MEVTP-3	0.9025	32.71	0.532	-4.30	0.29162525	43.0%
MEVTP-4	0.9893	33.94	0.532	-4.30	0.3013515	79.5%
MEVTP-5	0.9033	32.36	0.532	-4.30	0.2630995	44.0%
MEVTP-6	0.9466	33.96	0.532	-4.30	0.30405275	36.6%
MEVTP-7	0.9313	33.40	0.532	-4.30	0.3108545	43.2%
MEVTP-8	0.9646	34.42	0.532	-4.30	0.28331175	39.2%
MEVTP-9	0.9684	33.85	0.532	-4.30	0.30819275	50.0%
MEVTP-10	0.9425	37.59	0.527	-4.80	0.26392425	33.5%

Table S4 Predicted linear epitopes.

**Peptide**	**position**	**score**
PHLHFTIYKNKKKEQEARISWAAYIHVRAVEDVARLKIGGPGPGYMGAPGAGKAAYQ KIYVVEKNGGGNGVG	313-384	0.767
YPSYHSTPQRP	390-400	0.743
VSSEQSKAPHEKELYEKKYLYELYPRIAAYTSSTQ	135-169	0.693
AAYLQLFDTLSPEHRAEKGPGPGEQYRGTVGRAAYVK	192-228	0.683
TGVSLQQTPSGPGP	232-245	0.655
SGSSTTTD	247-254	0.596
NSARES	78-83	0.589
RSHGN	256-260	0.537
NPMNFIQPGAFKEIRLKELALDTNQLKSVPDGIFDRLTSLQKIWLHTNPWDCSCPRIDY LSRWLNKNSQKEQGSAKCSGSGKPVRSIICP	187-276	0.748
NKIQSIYCTDLRVLHQMPLLN	159-179	0.66
CEIQTIEDG	62-70	0.627
FNPLRHLGSYS	37-47	0.604
MELNFYKI	15-22	0.582
NPIQSLALGAFSG	86-98	0.558
IQSFKLPEYFSNLTN	136-150	0.551

Table S5 Predicted discontinuous epitopes.

Residues	Number of residues	score
A:T75, A:N78, A:S79, A:A80, A:R81, A:E82, A:S83, A:R85, A:A192, A:A193, A:L195, A:Q196, A:L197, A:F198, A:D199, A:T200, A:L201, A:S202, A:P203, A:E204, A:H205, A:R206, A:A207, A:E208, A:K209, A:G210, A:P211, A:G212, A:P213, A:G214, A:E215, A:Q216, A:Y217, A:R218, A:G219, A:T220, A:V221, A:G222, A:R223, A:A224, A:A225, A:Y226, A:V227, A:K228, A:S235, A:L236, A:Q237, A:Q238, A:P240, A:S241, A:G242, A:P243, A:G244, A:P245, A:G246, A:S247, A:G248, A:S249, A:S250, A:T251, A:T252, A:T253, A:H258, A:G259, A:N260, A:A261, A:P262, A:K263, A:K264, A:S266, A:F267, A:A270, A:G312, A:P313, A:H314, A:L315, A:H316, A:F317, A:T318, A:I319, A:Y320, A:N322, A:K323, A:K325, A:E326, A:Q327, A:E328, A:A329, A:R330, A:I331, A:S332, A:W333, A:A334, A:A335, A:Y336, A:I337, A:H338, A:V339, A:R340, A:A341, A:V342, A:E343, A:D344, A:V345, A:A346, A:R347, A:L348, A:K349, A:I350, A:G351, A:G352, A:P353, A:G354, A:P355, A:G356, A:Y357, A:M358, A:G359, A:A360, A:P361, A:G362, A:A363, A:G364, A:K365, A:A366, A:A367, A:Y368, A:Q369, A:I371, A:Y372, A:V373, A:V374, A:E375, A:K376, A:N377, A:G378, A:G379, A:G380, A:N381, A:G382, A:V383, A:F389, A:Y390, A:P391, A:S392, A:Y393, A:H394, A:S395, A:T396, A:P397, A:Q398	151	0.7

A:K141, A:A142, A:P143, A:H144, A:E145, A:K146, A:E147, A:L148, A:Y149, A:E150, A:K151, A:K152, A:Y153, A:L154, A:Y155, A:E156, A:L157, A:Y158, A:P159, A:I161, A:A162, A:A163, A:Y164, A:S166, A:S167, A:T168, A:T189, B:E1, B:N25	29	0.7
A:G1, A:I2, A:I3, A:T35, B:L112, B:A113, B:S114, B:E116, B:N117, B:G121, B:H122, B:L123, B:K124, B:L135, B:I136, B:Q137, B:S138, B:F139, B:K140, B:L141, B:P142, B:E143, B:Y144, B:S146, B:N147, B:L148, B:T149, B:N150, B:N159, B:K160, B:I161, B:Q162, B:S163, B:I164, B:Y165, B:C166, B:T167, B:D168, B:L169, B:R170, B:V171, B:L172, B:H173, B:Q174, B:M175, B:P176, B:L177, B:L178, B:N179, B:L180, B:S181, B:L182, B:L184, B:S185, B:L186, B:N187, B:P188, B:M189, B:N190, B:F191, B:I192, B:Q193, B:P194, B:G195, B:A196, B:F197, B:K198, B:E199, B:I200, B:R201, B:L202, B:K203, B:L205, B:L207, B:D208, B:T209, B:N210, B:Q211, B:L212, B:K213, B:S214, B:V215, B:P216, B:D217, B:G218, B:I219, B:F220, B:D221, B:R222, B:L223, B:T224, B:S225, B:L226, B:Q227, B:K228, B:I229, B:W230, B:L231, B:H232, B:T233, B:N234, B:P235, B:W236, B:D237, B:C238, B:S239, B:C240, B:P241, B:R242, B:I243, B:D244, B:Y245, B:L246, B:S247, B:R248, B:W249, B:L250, B:N251, B:K252, B:N253, B:S254, B:Q255, B:K256, B:E257, B:Q258, B:G259, B:S260, B:A261, B:K262, B:C263, B:S264, B:G265, B:S266, B:G267, B:K268, B:P269, B:V270, B:R271, B:S272, B:I273, B:I274, B:C275, B:P276	143	0.683
A:A132, A:V135, A:S136, A:S137, A:E138, A:Q139, A:S140	7	0.603

B:C14, B:L17, B:N18, B:F19, B:Y20, B:K21, B:L35, B:S36, B:N38, B:P39, B:L40, B:R41, B:H42, B:L43, B:G44, B:S45, B:Y46, B:S47, B:C62, B:E63, B:I64, B:Q65, B:T66, B:I67, B:E68, B:D69, B:G70, B:Q73, B:N86, B:P87, B:I88, B:Q89, B:S90, B:L91, B:A92, B:L93, B:G94, B:A95, B:F96, B:S97, B:G98, B:N111, B:F118, B:P119, B:I120	45	0.569
A:D254, A:R256, A:S257	3	0.525

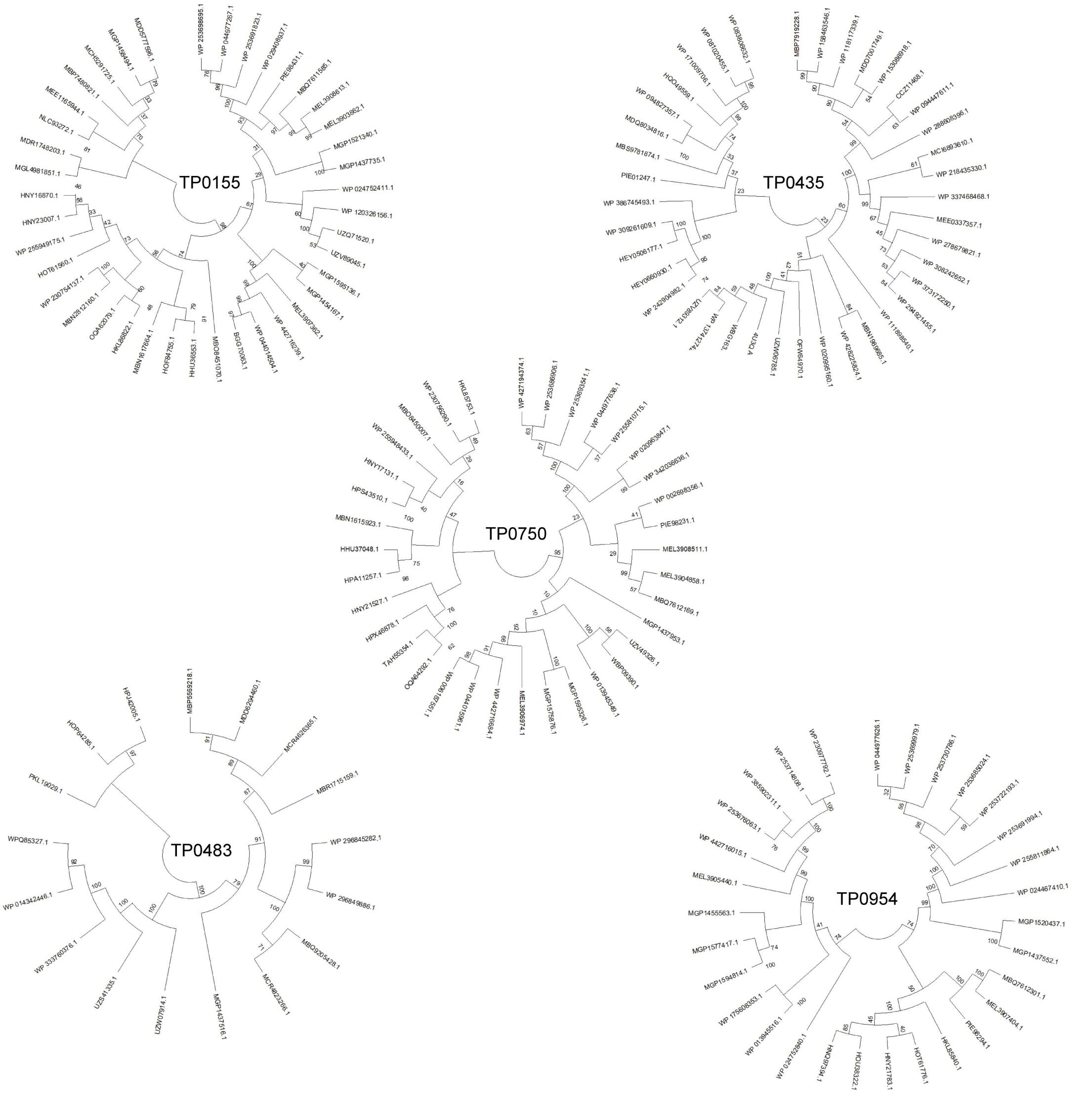

Fig. S1. Phylogenetic analysis of target *Treponema pallidum* using MEGA software. At each branch the bootstrap values (1–100) are also indicated.

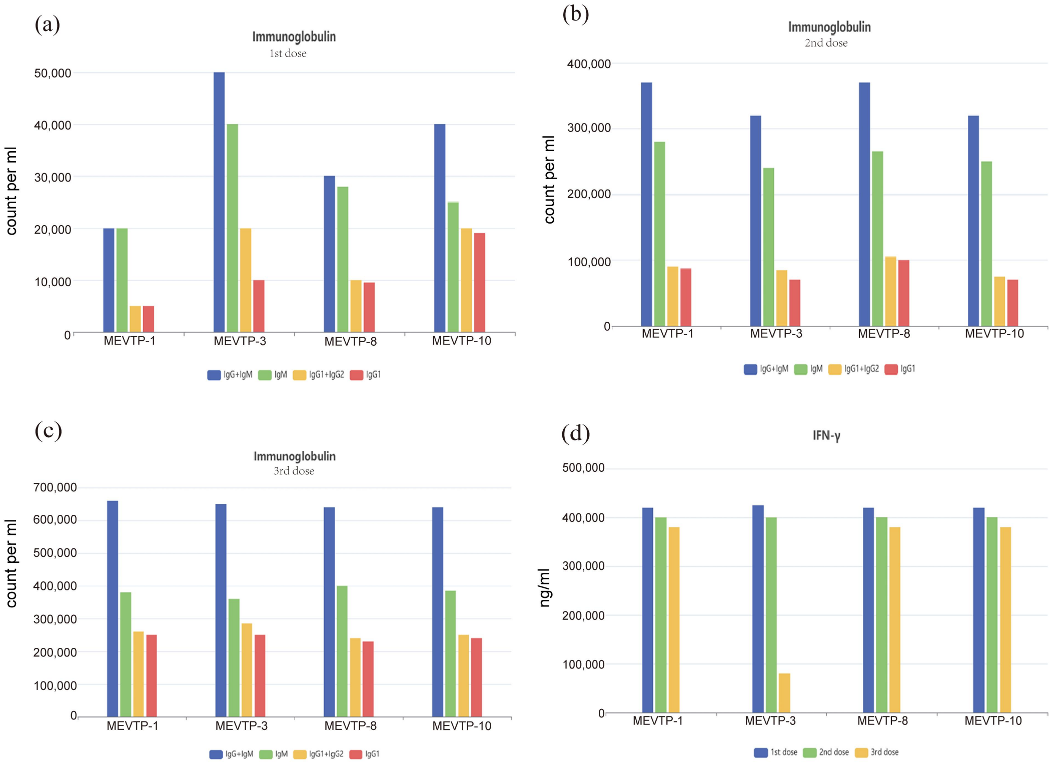

Fig. S2. Evaluation in the C-IMMSIM server. (a-c) The figures show changes in immunoglobulins after the first, second, and third vaccine doses, respectively. Results indicate that immunoglobulin levels significantly increased with the number of doses, demonstrating good immunogenicity of the vaccine. **(d)** The figure presents IFN-γ measurement results after three doses in each vaccine group. The significant rise in IFN-γ values further confirms the immune activation effect of the vaccine.
